# Mechanism of Tubulin Oligomers and Single-Ring Disassembly Catastrophe

**DOI:** 10.1021/acs.jpclett.2c00947

**Published:** 2022-06-07

**Authors:** Asaf Shemesh, Avi Ginsburg, Raviv Dharan, Yael Levi-Kalisman, Israel Ringel, Uri Raviv

**Affiliations:** † Institute of Chemistry, The Hebrew University of Jerusalem, Jerusalem 9190401, Israel; ‡ Center for Nanoscience and Nanotechnology, The Hebrew University of Jerusalem, Jerusalem 9190401, Israel; § Institute of Life Sciences, The Hebrew University of Jerusalem, Jerusalem 9190401, Israel; ∥ Institute for Drug Research, School of Pharmacy, The Hebrew University of Jerusalem, Jerusalem 9112102, Israel

## Abstract

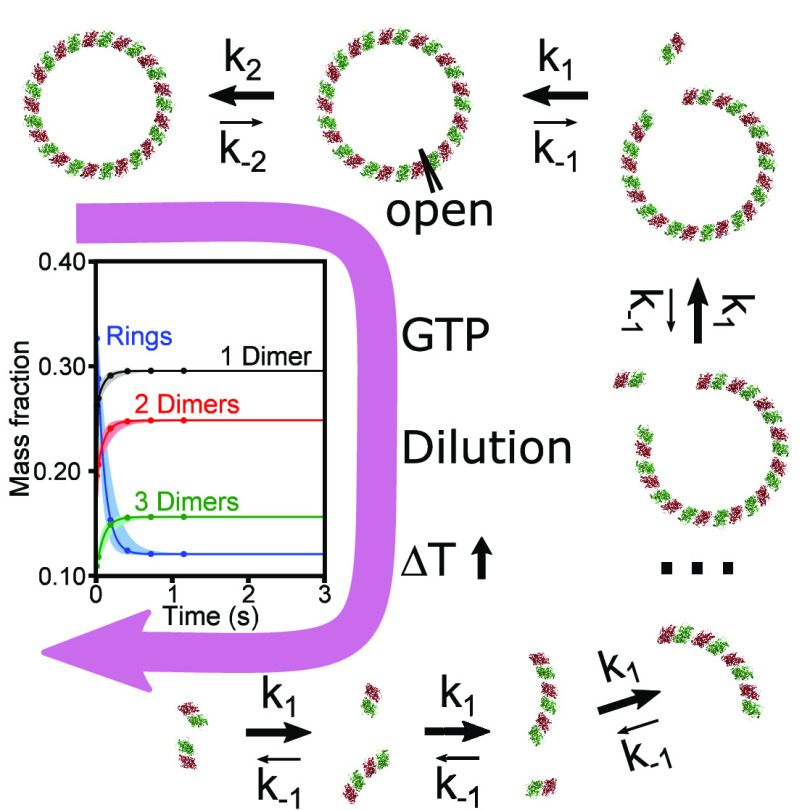

Cold tubulin dimers coexist with tubulin oligomers and single rings. These structures are involved in microtubule assembly; however, their dynamics are poorly understood. Using state-of-the-art solution synchrotron time-resolved small-angle X-ray scattering, we discovered a disassembly catastrophe (half-life of ∼0.1 s) of tubulin rings and oligomers upon dilution or addition of guanosine triphosphate. A slower disassembly (half-life of ∼38 s) was observed following an increase in temperature. Our analysis showed that the assembly and disassembly processes were consistent with an isodesmic mechanism, involving a sequence of reversible reactions in which dimers were rapidly added or removed one at a time, terminated by a 2 order-of-magnitude slower ring-closing/opening step. We revealed how assembly conditions varied the mass fraction of tubulin in each of the coexisting structures, the rate constants, and the standard Helmholtz free energies for closing a ring and for longitudinal dimer–dimer associations.

Microtubules (MTs) are filamentous protein nanotubes (25 nm in diameter) with walls comprised of assembled protofilaments, built from tubulin dimers. MTs are involved in vital cellular processes, including cell division and intracellular trafficking. A growing MT can abruptly shrink even when there is plenty of free tubulin in the solution, in a process called catastrophe.^1^ Moreover, while some MTs may completely disassemble, others may continue to grow. The activity and stability of MT, its dynamic assembly and disassembly processes, mostly depend on whether guanosine triphosphate (GTP) or guanosine diphosphate (GDP) molecules are bound to the tubulin dimers and are determined by the GTPase activity of tubulin.^1−5^

GTP binds tubulin in two sites, a non-exchangeable (N) site and an exchangeable (E) site. α-Tubulin binds GTP that is buried at the monomer–monomer interface (N-site), whereas β-tubulin binds GTP that is exposed on the monomer surface (E-site) and can be readily hydrolyzed into GDP upon MT polymerization, predominantly at the interface with GDP-tubulin.^3^

The tubulin dimer with GTP at the E-site (GTP-tubulin) can initiate and promote protofilaments and MT assembly.^3,6−10^ The tubulin dimer with GDP at the E-site (GDP-tubulin) assumes a kinked conformation between its α- and β-tubulin subunits.^7,8,11^ Recent studies suggest that in the soluble state, both GDP- and GTP-tubulin adopt, on average, similar kinked conformations and straightening occurs when GTP-tubulin polymerizes into MT.^11−14^

GDP bound to tubulin cannot be exchanged back to GTP as long as the dimer is a part of the MT polymeric form.^2^ The assembled GDP-tubulin (whose E-site is now buried within the polymer) is under conformational tension that can promote MT disassembly catastrophe.^1,7,8,11,15^ The protofilaments that are disassembling from MT edges are curving outward and were shown to bend in a direction perpendicular to the curvature plane of the polymeric tube, suggesting a specifically curved dimer symmetry.^16^ The resulting disassembled curved GDP-rich tubulin dimers and curved one-dimensional (1D) tubulin oligomers can promote the assembly of tubulin single rings (38 nm in diameter) or double rings.^3,12,17−21^ Free GDP-tubulin dimers may readily exchange their bound GDP for GTP.^22^ However, the GDP to GTP exchange reaction equilibrium constant is an order of magnitude smaller for dimers whose E-sites are buried (between two other dimers) in oligomers or rings.^23^

Tubulin oligomers and single rings are dynamic structures that coexist with MTs and involved in MT assembly and disassembly processes.^16−20,24^ The rings are a storage form of active tubulin subunits.^24^ The initial phase of MT assembly is accompanied by a simultaneous ring disassembly,^17^ providing most (85–90%) of the tubulin subunits incorporated in the initial stages of MT assembly.^18^ During MT disassembly, the increase in the concentration of rings is delayed until the concentration of dimers is sufficiently high.^17^ Despite their involvement in MT assembly, the mechanism of tubulin-ring assembly and disassembly remained poorly understood.

MT assembles from solutions of ice-cold tubulin only after the temperature is increased and GTP is available. Ice-cold tubulin solutions are rich in dimers, oligomers, and rings, which disassemble at the onset of MT assembly. It is therefore of utmost importance to resolve the mechanism of ring assembly and disassembly when GTP is added or the temperature is increased. We have therefore analyzed the structure, interactions, and kinetics of purified^25^ cold GDP-tubulin and cold GTP-tubulin solutions using solution small-angle X-ray scattering (SAXS).^26^ Steady-state SAXS^27−30^ data were analyzed using atomic structural models of tubulin assemblies^15,31−33^ whose mass fraction was determined on the basis of an isodesmic thermodynamic model of tubulin self-association. The analyses revealed the structure and mass fractions of the tubulin dimer, tubulin single rings, and tubulin oligomers (ring fragments), as a function of the total tubulin concentration. Additionally, we determined the longitudinal association standard Helmholtz free energies between GTP- or GDP-tubulin dimers in cold solutions. Using time-resolved SAXS,^34,35^ we analyzed the disassembly kinetics of GDP-tubulin single rings upon dilution, GTP addition, or a temperature jump. We discovered a rapid isodesmic disassembly catastrophe (half-life of ∼0.1 s) of cold GDP-tubulin single rings upon addition of GTP or sample dilution. A slower ring isodesmic dissociation kinetics (half-life of ∼38 s) was induced by a temperature increase.

Cryo-transmission electron microscopy (cryo-TEM) images (Figure S1) reveal that free tubulin dimers coexisted with tubulin single rings and small tubulin oligomers in curved conformations (ring fragments). In excess GTP, the fraction of tubulin single rings was smaller than in excess GDP.^17,36^ High-performance liquid chromatography (HPLC) analysis showed that seven heating–cooling cycles (see section S1 of the Supporting Information) or incubation in 10 ± 0.5 mM GDP led to 94% GDP-tubulin and 6% GTP-tubulin at the E-site.^23^ After seven heating–cooling cycles, tubulin retains its MT assembly capability,^23^ though the fraction of tubulin single rings was larger than after incubation in 10 ± 0.5 mM GDP. The excess of rings is most likely kinetically trapped (stable) rings. A small fraction of stable tubulin single rings remains even after SEC elution experiments,^36^ suggesting that even after ∼100-fold dilution, some of the rings do not completely disassemble.

Steady-state SAXS measurements at different concentrations of GDP- and GTP-tubulin (Figure 1) were performed below the critical temperature for MT assembly. The two data sets were fitted to a linear combination of tubulin single rings and oligomers (ring fragments), whose mass fractions were based on a thermodynamic model of tubulin self-association (eqs S1 and S6). The best fit was obtained after assuming that 10% of the tubulin mass fraction formed kinetically trapped stable tubulin single rings. The rest of the tubulin mass was distributed according to the thermodynamic model (eqs S4–S6). At high GDP-tubulin concentrations, the mass fraction of dimers in tetramers was higher than the mass fraction of free dimers (Figure 1C). At the concentration scale, however, the concentration of free dimers was higher than the concentration of tetramers, as expected.^37^ In a separate experiment, a GDP-tubulin sample at a higher concentration adequately fitted the same model and further supported these results (Figure S3).

**Figure 1:**
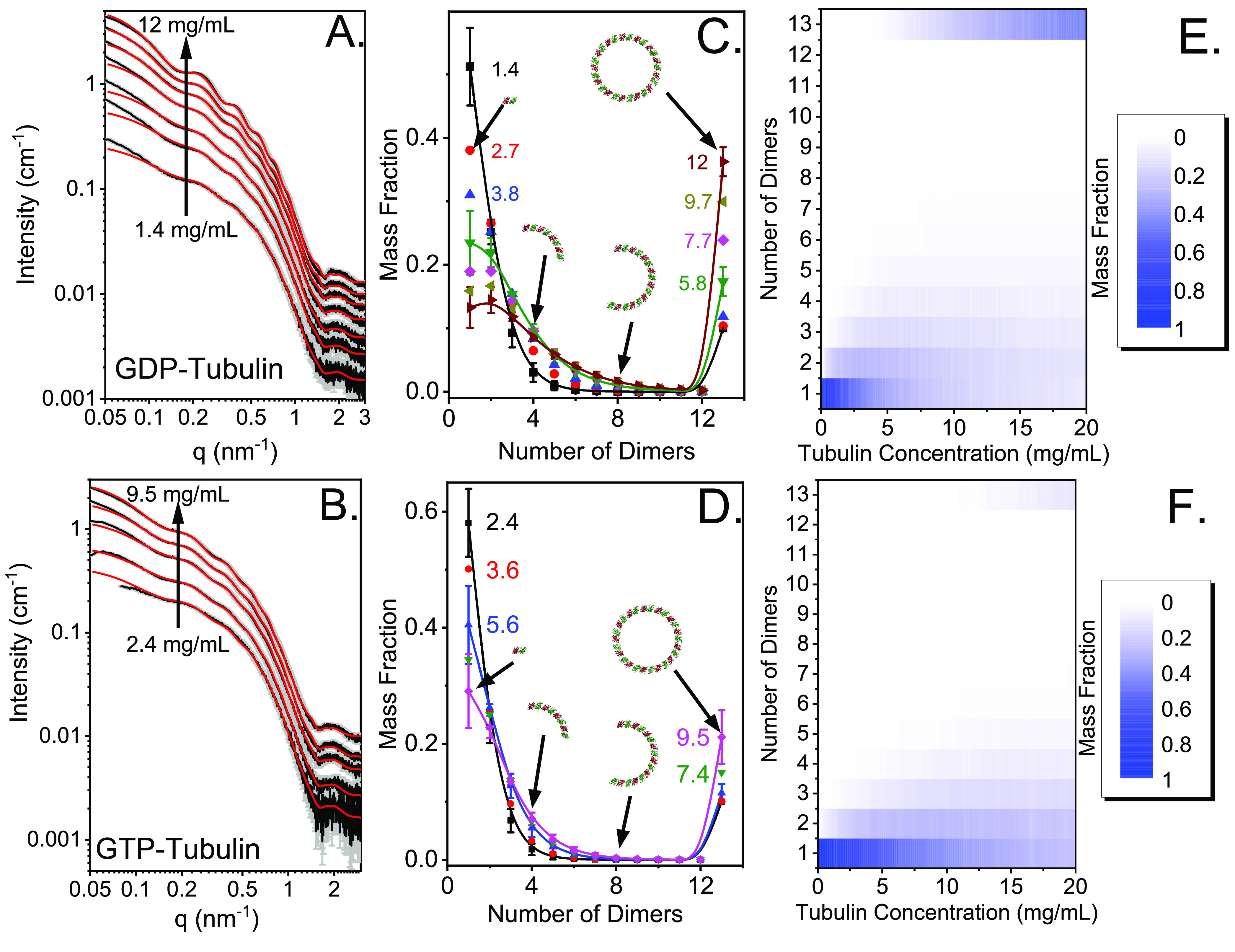
Steady-state analysis of GDP- and GTP-tubulin solutions at 9 °C. Azimuthally integrated background-subtracted^38,42,43^ absolute scattering intensities^44^ as a function of *q*, the magnitude of the scattering vector (black curves, gray error bars) from (A) a GDP-tubulin solution following seven heating–cooling cycles and (B) a GTP-tubulin solution after dilution series, as indicated. The data were fitted (red curves) to a linear combination of computed scattering curves, using D+ software (https://scholars.huji.ac.il/uriraviv/software/d-software),^31,32^ based on atomic models of tubulin rings and oligomers (see Figure S2 and subsection SAXS Models in section S1 of the Supporting Information). The mass fraction of each structure was determined by a thermodynamic model of tubulin self-association (eqs S1 and S6), according to the best-fit standard Helmholtz free energies, presented in Table 1. The measured intensity at *q* < 0.08 nm^–1^ of 2.4 mg/mL GTP-tubulin was omitted owing to a technical measurement error. Mass fraction distributions of (C) GDP-tubulin and (D) GTP-tubulin rings and oligomers (ring fragments) as a function of size (number of tubulin dimers), based on the standard Helmholtz free energies that best fit the data (eq S7) and the measured tubulin concentration (indicated in units of milligrams per milliliter). The mass fraction of 13 dimers includes open, closed, and stable rings. The contribution of open rings was negligible, and the contribution of stable rings was typically 10% of the total tubulin mass fraction. The remaining mass fraction of 13 dimers was due to closed rings, as predicted by the thermodynamic model. Heat maps of the (E) GDP-tubulin and (F) GTP-tubulin mass fraction, plotted in the plane of the number of dimers in assembly vs the total tubulin concentration, computed according to eq S1, using the parameters from Table 1. Data were measured at the P12 EMBL BioSAXS Beamline in PETRA III (DESY, Hamburg, Germany).^29,30^

Panels C and D of Figure 1 show the distribution of tubulin oligomers (ring fragments) that best fit the data at each GDP- and GTP-tubulin concentration, respectively. The best-fit standard Helmholtz dimer–dimer association free energy per longitudinal contact, *ΔF*_c_^°^, and standard Helmholtz free energy cost for closing a curved oligomer into a ring, *ΔF*_RC_^°^, are listed in Table 1. As explained,^36,38,39^ the relation between *ΔF*_c_^°^, on the molar fraction scale, and the standard Gibbs free energy for dimer–dimer self-association, *ΔG*_c_^°^, on the concentration scale, is Δ*G*_c_^°^ ≈ *ΔF*_c_^°^ + 4*k*_B_*T*.

**Table 1:** Best-Fit Thermodynamic Parameters Used to Analyze the SAXS Data^a^

	*T* (°C)	*ΔF*_c_^°^ (*k*_B_*T*)	*ΔF*_RC_^°^ (*k*_B_*T*)	*ΔF*_c_^°^ (kcal mol^–1^)	*ΔF*_RC_^°^ (kcal mol^–1^)
GDP-tubulin	9	–14.6 ± 0.3	9 ± 1	8.2 ± 0.2	5.2 ± 0.6
GTP-tubulin	9	–13.7 ± 0.3	6 ± 1	7.7 ± 0.2	3.9 ± 0.6

a The standard Helmholtz free energies were obtained on the molar fraction scale. The standard Gibbs free energies on the concentration scale can be obtained by adding 4 *k*_B_*T*.^36^

GTP-tubulin had weaker standard Helmholtz free energy values, suggesting that GTP acts as a hydrotrope that increases the solubility of tubulin, like ATP.^40,41^ The thermodynamic analysis is applicable for GDP-tubulin, where there is no hydrolysis reaction. The fact that the same analysis explained the data of cold GTP-tubulin solutions (after adjusting the dimer–dimer association energy) is consistent with our recent HPLC experiments that showed that at low temperatures the hydrolysis of GTP is very slow, when free in the buffer or bound to the tubulin dimers.^23^

Time-resolved SAXS (TR-SAXS) measurements^17,45−47^ followed the tubulin-ring disassembly kinetics, triggered by either dilution (Figure 2), GTP addition (Figure 3), or a temperature jump (Figure 4). GTP addition includes dilution of the tubulin solution; hence, it was important to separate the effect of dilution from the effect of addition of GTP. The dilution and GTP addition experiments also simulated the dilution and nucleotide exchange reactions that may occur during SEC elution experiments.^23,36^

**Figure 2:**
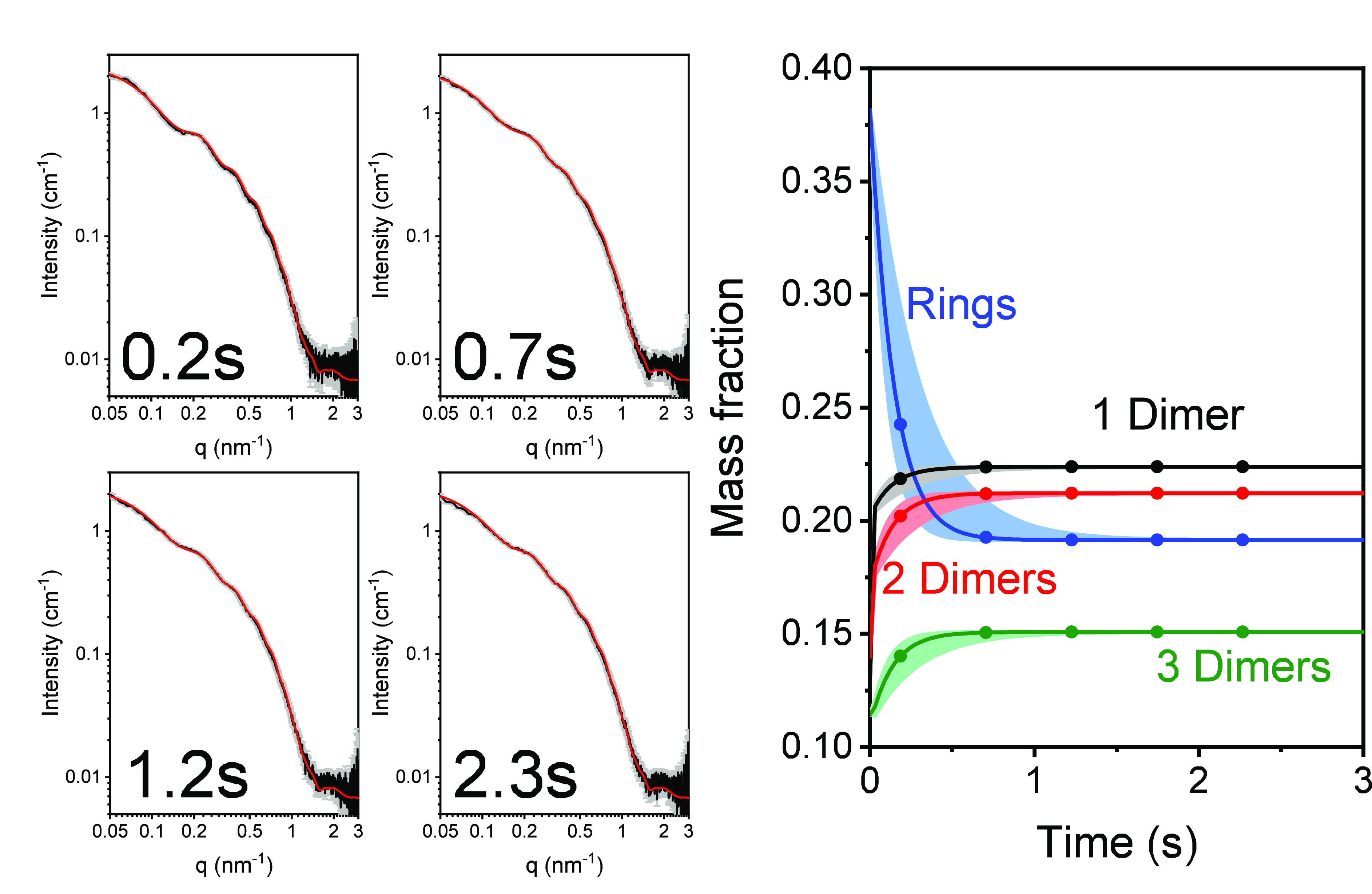
GDP-tubulin single-ring disassembly catastrophe following dilution. GDP-tubulin (obtained by seven heating–cooling cycles) was mixed in a stopped-flow setup with BRB80, supplemented with 0.7 ± 0.1 mM GDP, as explained in subsection TR-SAXS Measurement Protocol and Analysis in section S1. The four panels on the left present examples of TR-SAXS data (black curves, gray error bars) at selected time points, as indicated. The complete TR-SAXS data set is presented in Figure S6. The data were fit (red curves) to our kinetic model (eqs S12 and S13). The best-fit model parameters (Table 2) determined the mass fraction of rings and oligomers (ring fragments) as a function of time (right panel), which in turn were used to compute the red curves, using eq S6. The errors in the mass fractions (right panel) are indicated by shaded colored areas, surrounding the solid curves. Data were measured at the ID02 beamline (ESRF, Grenoble, France).^48^

**Figure 3:**
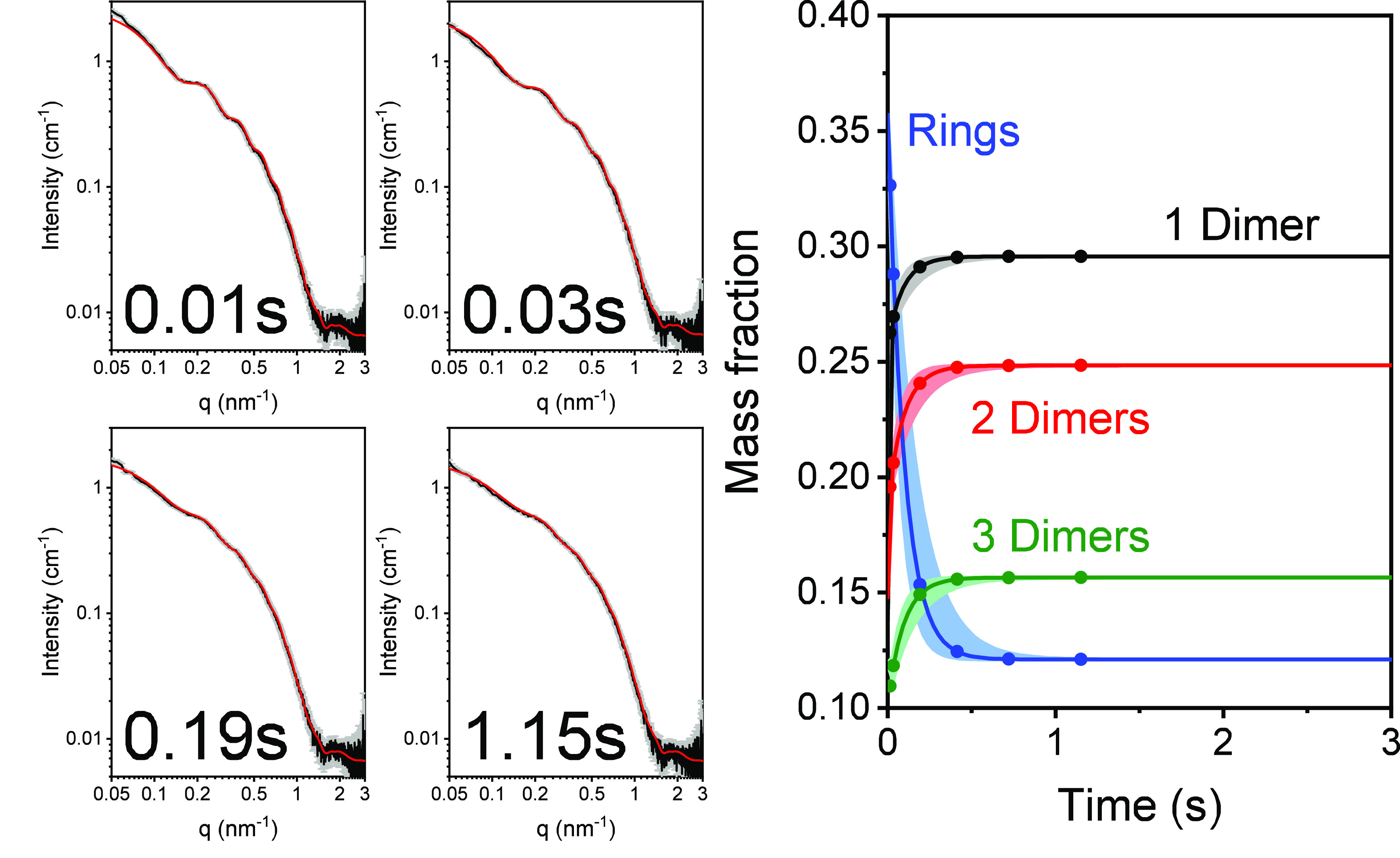
GDP-tubulin single-ring disassembly catastrophe following GTP addition. GDP-tubulin (obtained by seven heating–cooling cycles) was mixed in a stopped-flow setup with BRB80, supplemented with 0.7 ± 0.1 mM GDP and 8 ± 0.5 mM GTP, as explained in subsection TR-SAXS Measurement Protocol and Analysis in section S1. The four panels on the left present examples of TR-SAXS data (black curves, gray error bars) at selected time points, as indicated. The complete TR-SAXS data set is presented in Figure S7. The data were fit (red curves) to our kinetic model (eqs S12 and S13). The right panel shows the mass fraction of rings and ring fragments as a function of time. Table 2 shows the best-fit model parameters. Data were measured at the ID02 beamline (ESRF).^48^

**Figure 4:**
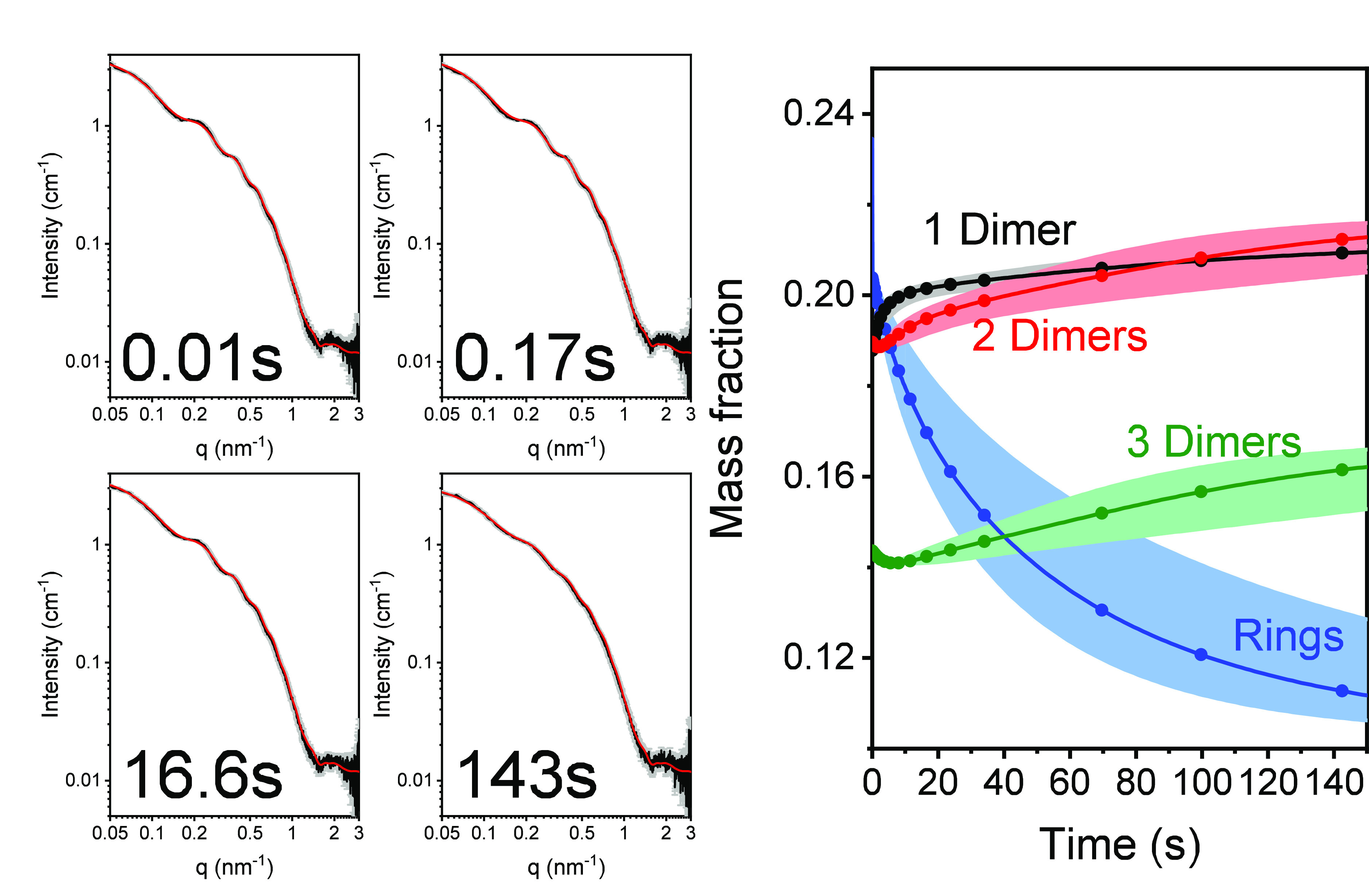
GDP-tubulin single-ring disassembly following a temperature jump. GDP-tubulin (obtained by seven heating–cooling cycles) at 9 °C was injected by a stopped-flow setup into a quartz capillary, kept at 36 °C, as explained in subsection TR-SAXS Measurement Protocol and Analysis in S1. The four panels on the left present examples of TR-SAXS data (black curves, gray error bars) at selected time points, as indicated. The complete TR-SAXS data set is presented in Figures S8 and S9. The data were fitted (red curves) to our kinetic model (eqs S12 and S13). The right panel shows the mass fraction of rings and ring fragments as a function of time. Table 2 shows the best-fit model parameters. Data were measured at the ID02 beamline (ESRF).^48^

The TR-SAXS data were fit to an isodesmic kinetic model (eqs S12 and S13), in which dimers were rapidly added or removed one at a time and rings were closed or opened at a rate that was 2 orders of magnitude slower (Table 2). The thermodynamic parameters determined by the steady-state measurements (Table 1) and SEC-SAXS chromatogram analysis^36^ were used to estimate the initial size distribution and to calculate the ratio between the assembly and disassembly rate constants, according to the detailed balance conditions (eqs S10 and S11). The rate constants and the standard Helmholtz free energies used to analyze the TR-SAXS data are summarized in Table 2.

**Table 2:** Best-Fit Rate Constants and Their Associated Thermodynamic Parameters Used to Analyze the TR-SAXS Data^a^

	*T* (°C)	*k*_1_ (M^–1^ s^–1^)	*k*_–1_ (s^–1^)	*k*_2_ (s^–1^)	*k*_–2_ (s^–1^)	*ΔF*_c_^°^ (k_B_T/kcal mol^–1^)	*ΔF*_RC_^°^ (k_B_T/kcal mol^–1^)
dilution	9	(9 ± 5) × 10^7^	3000 ± 2000	≥1 × 10^4^	≥40	–14.5 ± 0.2/–8.1 ± 0.1	9 ± 1/5 ± 0.5
addition of GTP	9	(9 ± 4) × 10^7^	3000 ± 1000	≥1 × 10^4^	≥40	–14.2 ± 0.2/–8 ± 0.1	9 ± 1/5 ± 0.5
temperature change	9–36	(4 ± 2) × 10^3^	0.2 ± 0.1	≥10	≥3	–14.2 ± 0.2/–8.7 ± 0.1	13 ± 2/8 ± 1

a The standard Helmholtz free energies were obtained on the molar fraction scale. The standard Gibbs free energies on the concentration scale can be obtained by adding 4 *k*_B_*T*.^36^ A rate constant that was 2 orders of magnitude larger for ring closure did not change the fitting results, suggesting that our data were insensitive to these rate constants.

Very rapid (half-life of ∼0.1 s) ring and oligomer disassembly catastrophe kinetics were observed upon dilution or GTP addition. Steady state were attained within ∼1 s (Figure S5). The observed disassembly products were dimers, tetramers (dimer of dimers), and hexamers (trimer of dimers). Larger oligomers did not accumulate to detectable amounts. The fraction of hexamers at the steady state was similar upon dilution or GTP addition. Upon dilution, however, the fraction of rings was higher, and consequently, the fractions of dimers and tetramers were lower.

The fraction of tubulin rings decreases with an increase in temperature.^17,24^ We observed, however, a slower GDP-tubulin ring disassembly rate (half-life of ∼38 s) following a temperature jump (Figure 4). Initially, the net amount of tetramers and hexamers decreased because they disassembled at 36 °C; however, then the rings continued to disassemble, and hence, the mass fraction of tetramers and hexamers increased. Furthermore, the mass fractions of tubulin in free dimers and tetramers were comparable after ≈40 s (unlike the low-temperature results), suggesting larger oligomers were more stable at a higher temperature. van’t Hoff analysis, based on the standard self-association free energies at 9 and 36 °C (Tables 1 and 2), estimates that upon tubulin self-association the standard entropy increased (by ≈12 ± 10 cal K^–1^ mol^–1^), suggesting that water molecules were released upon association and increased the entropy of GDP-tubulin oligomerization.^49^

Similar steady-state results were obtained from a 1 h incubation of GDP-tubulin at 36 °C, where a strong longitudinal association standard Helmholtz free energy (*ΔF*_c_^°^ = −16.2 ± 1 *k*_B_*T* or −9.9 ± 0.6 kcal mol^–1^) was fit to the data (Figure S4). The data in Figure S4, however, included those of tubulin aggregates; hence, the fit was limited to a smaller *q* range (0.2 nm^–1^ ≤ *q* ≤ 3 nm^–1^), and its precision was somewhat lower. The characteristic oscillation pattern of ring and ring fragments was substantially reduced compared with that of the 9 °C scattering curves (Figure 1). We attribute the change to the increased flexibility and thermal perturbations caused by the increase in temperature. An earlier study also found that above ≈25 °C the fraction of tubulin rings at the steady state decreases with an increase in temperature.^18^ In our earlier study,^17^ we showed that a similar decrease in the fraction of GTP-tubulin single rings was observed after the temperature was increased.

Tubulin double rings were identified as the depolymerization product of purified MT caused by low temperatures.^20,21^ The self-association of cold GDP-tubulin into double rings was examined under an excess of 7 mM MgCl_2_ using velocity sedimentation measurements at increasing tubulin concentrations. The observations were described in terms of a thermodynamic model of isodesmic self-association, revealing a longitudinal standard association Helmholtz free energy, *ΔF*_c_^°^, of approximately −7.8 kcal mol^–1^.^50,51^ TEM and SAXS measurements resolved the structure of the GDP-tubulin double rings.^50,52^ TR-SAXS showed that the double rings were destabilized within ≈1 min after the temperature was increased to 37 °C or when GTP was added.^20,53^

In this paper, we examined the disassembly catastrophe mechanism of tubulin oligomers and single rings, involved in the early steps of microtubule assembly. We showed that at low temperatures and over a wide range of GDP- and GTP-tubulin concentrations, the entire distribution of tubulin single rings and one-dimensional curved oligomers (ring fragments) is consistent with a thermodynamic theory of isodesmic tubulin self-association. GTP acts as an effective hydrotrope that increases the solubility of tubulin, reduces the longitudinal dimer–dimer Helmholtz standard association free energy, and reduces the free energy of ring closure. Therefore, solutions of GTP-tubulin contained higher concentrations of tubulin dimers and smaller assemblies, compared with those of the corresponding GDP-tubulin solutions. GDP-tubulin single rings rapidly destabilized upon dilution or GTP addition. Time-resolved experiments illuminated ring disassembly catastrophe (half-life of ∼0.1 s) and were consistent with an isodesmic disassembly mechanism, involving ring opening followed by consecutive single-dimer removal steps that were 2 orders of magnitude faster. A similar disassembly mechanism explained the disassembly of cold GDP-tubulin rings following a temperature jump to 36 °C, however, at a significantly slower rate (half-life of ∼38 s).

## Supporting Information Available

Materials and methods, cryo-TEM images, SAXS models of rings and ring fragments, additional GDP-tubulin data, steady state after ring disassembly catastrophe, tubulin single-ring catastrophe following dilution, tubulin single-ring catastrophe following GTP addition, and tubulin single-ring disassembly following a temperature increase (PDF)
Transparent Peer Review report available (PDF)
