# Supplementary material for: Mechanism of Tubulin Oligomers and Single-Ring Disassembly Catastrophe
Source: J Phys Chem Lett. 2022 Jun 7;13(23):5246–52. doi: 10.1021/acs.jpclett.2c00947 (PMC9208022; doi:10.1021/acs.jpclett.2c00947)
Supplement: Supplementary file 1 — jz2c00947_si_001.pdf [file jz2c00947_si_001.pdf]

Supporting Information
Mechanism of Tubulin Oligomers and Single-Rings Disassembly Catastrophe

Asaf Shemesh,[†,‡] Avi Ginsburg,[†] Raviv Dharan,[†] Yael Levi-Kalisman,[¶,‡] Israel Ringel,[§] and Uri Raviv[*,†,‡]

†*Institute of Chemistry, The Hebrew University of Jerusalem, Jerusalem, 9190401, Israel*

‡*Center for Nanoscience and Nanotechnology, The Hebrew University of Jerusalem, Jerusalem, 9190401, Israel*

¶*Institute of Life Sciences, The Hebrew University of Jerusalem, Jerusalem, 9190401, Israel*

§*Institute for Drug Research, School of Pharmacy, The Hebrew University of Jerusalem, 9112102, Jerusalem, Israel*

E-mail: uri.raviv@mail.huji.ac.il

Phone: +972 (2) 6586030. Fax: +972 (2) 5660425

Supporting Information Available

S1. Materials and Methods

Materials were purchased from Sigma-Aldrich Co. The accuracy of the reported temperatures was ±1 °C.

Tubulin Purification

Tubulin was purified from porcine brains, using a modified version of the high-molarity buffer purification method,[1] as described earlier.[2] Purified tubulin was in BRB80 (80 mM 1,4-piperazineediethanesulfonic acid (PIPES), 1 mM ethylene glycol-bis(β-aminoethyl ether)-N,N,N',N'-tetraacetic acid (EGTA), 1 mM $MgCl_2$, at pH 6.9 (adjusted with KOH)). Immediately following purification, aliquots were flash-frozen in liquid nitrogen, stored at −80 °C, and used within the following two weeks.[3,4] Before used in experiments, tubulin was thawed on ice.

Heat-Cool Cycles

To hydrolyze the residual GTP, seven heat-cool cycles were applied, which led to tubulin-bound nucleotide compositions at the E-site of 94% GDP-tubulin, and 6% GTP-tubulin.[5] We verified that this tubulin retained its activity and was able to form MTs to a similar extent before and after seven heat-cool cycles.[5,6]

Similar nucleotide composition was achieved by incubation of fresh tubulin samples (i.e. a sample that did not undergo seven heat-cool cycles) in the presence of 10 ± 0.5 mM GDP.[7]

Sample Preparations

Before any measurement, the tubulin samples were spun-down for 30 min at 4 °C and 20,800 g. The top 60%, taken from the supernatant, was then used for the experiments.

Steady-state SAXS

42 ± 3 mg/mL purified tubulin, containing a total GTP and GDP concentration of 1.3 ± 0.1 mM and 0.9 ± 0.1 mM ATP, was used for the steady-state SAXS experiments. GTP-tubulin was obtained by diluting the tubulin sample in BRB80 and adding 3 ± 0.1 mM GTP. GDP-tubulin was obtained by applying seven heat-cool cycles, diluting the sample in BRB80, and adding 3 ± 0.1 mM GDP. Steady-state SAXS measurements were performed at 9 °C.

Time-resolved SAXS

24 ± 2 mg/mL tubulin samples, containing a total GTP and GDP concentration of 0.7 ± 0.1 mM and 0.4 ± 0.05 mM ATP, underwent seven additional heat-cool cycles and spin-down as described above. Samples were then transferred to the stopped-flow reservoir, kept at 9 °C, from which they were injected into the stopped-flow quartz capillary, kept at either 9 °C or 36 °C.

Cryo-TEM

GTP- and GDP-tubulin. 36 ± 3 mg/mL purified tubulin, containing 0.6 ± 0.1 mM GTP, 0.4 ± 0.05 mM GDP, and 0.7 ± 0.1 mM ATP, were thawed on ice and spun-down as described above. Either 10 ± 0.5 mM GTP or 10 ± 0.5 mM GDP were added, and the samples were incubated on ice for 1.5 h. The incubated samples were then 10-fold diluted in BRB80 supplemented with either 1 ± 0.1 mM GTP or 1 ± 0.1 mM GDP, incubated on ice for 20 min, and flash-frozen.

Cycled tubulin. 44 ± 4 mg/mL purified tubulin, containing a total GTP and GDP concentration of 1.3 ± 0.1 mM and 0.7 ± 0.1 mM ATP, underwent seven additional heat-cool cycles and spun down, as described above. The supernatant was then incubated for 2 h on ice, and 10-fold diluted in BRB80, supplemented with 1.3 ± 0.1 mM GDP. 1 h after the dilution, the samples were flash-frozen.

Solution SAXS and Time-Resolved SAXS Measurements and Data Reduction

SAXS experiments were performed at P12 EMBL BioSAXS Beamline (headed by D. Svergun) in PETRA III (DESY, Hamburg),[8] and in ID02 beamline (headed by T. Narayanan) in the European synchrotron radiation facility (ESRF, Grenoble).[9] At P12, measurements were taken using an automated temperature controlled sample changer setup.[10] Samples were injected into a temperature controlled 2 mm quartz capillary. The sample-to-detector distance was 3 m, resulting in a q-range of $0.05 - 5\,\mathrm{nm}^{-1}$. The size of the beam was $0.2\times0.12\,\mathrm{mm}^2$ and the exposure time per frame was 45 ms. The X-ray wavelength was 1.24 Å, at a flux of $5 \times 10^{12}\,\mathrm{photons}\cdot\mathrm{s}^{-1}$, and the scattered intensity was recorded on a Dectris Pilatus 2M detector.

At ID02, time-resolved SAXS (TR-SAXS) data were measured using a BioLogic SFM-400 stopped-flow apparatus, as explained in our earlier publications.[11–13] The capillary outer diameter was 1.4 mm, and the sample-to-detector distance was 2.5 m, resulting in a q-range of $0.03 - 3\,\mathrm{nm}^{-1}$. The size of the beam was $0.3\times0.2\,\mathrm{mm}^2$ and the exposure time per frame was 20 ms. The intervals between frames was 160 ms or longer. The X-ray wavelength was 0.995 Å, at a flux of $1 \times 10^{13}\,\mathrm{photons}\cdot\mathrm{s}^{-1}$, and the scattered intensity was recorded on a Rayonix MX170-HS detector.[9,14]

The 2D scattering images were normalized to the intensity of the transmitted beam and azimuthally averaged to yield the scattering intensity as a function of the magnitude of the scattering vector, q, using the integrated analysis pipeline SASFLOW[15] at the P12 beamline, and the online SAXS/WAXS data reduction package (SPD)[16] at ID02.

Background measurements, before and after each sample, were performed from the solvent of each sample, under identical measurement conditions. Background scattering curves were averaged and subtracted from the averaged sample signal, resulting in the final background-subtracted scattering intensity curve, as explained in our earlier papers.[2,17–19] Absolute intensity scales were obtained using water, whose differential cross section is $0.0164\,\mathrm{cm}^{-1}$,

when $1 < q < 4\,\mathrm{nm}^{-1}$.[20]

TR-SAXS Measurement Protocol and Analysis

The disassembly kinetics following dilution were measured by mixing 100 μL of 13 ± 1 mg/mL GDP-tubulin (following seven heat-cool cycles) and 100 μL of BRB80, supplemented with 0.7 ± 0.1 mM GDP (GDP was added to the diluting solution to maintain a constant concentration of unbound GDP in the sample). The disassembly kinetics following GTP addition, was measured by mixing 100 μL of 13 ± 1 mg/mL GDP-tubulin and 100 μL of BRB80, supplemented with 0.7 ± 0.1 mM GDP and 8 ± 0.5 mM GTP. Both experiments were done at 9 °C. The kinetics following the temperature jump was performed in a stopped-flow setup by injecting 200 μL of the GDP-tubulin sample, kept at 9 °C, into the preheated stopped-flow capillary, kept at 36 °C. The temperature jump was relatively instantaneous compared with the entire kinetics.

k_{-1} and k_{-2} were fitted to the TR-SAXS data (using Equations S12 and S13)), whereas k_1 and k_2 where calculated based on the detailed balance conditions (Equations S10 and S11), using thermodynamic parameters close to that of the steady-state data analysis (see Table 2).

Cryo-TEM

Tubulin solutions were directly imaged using transmission electron microscopy at cryogenic temperatures (cryo-TEM). A droplet of 3 μL was deposited on a 300 mesh Cu Lacey grid (Ted Pella Ltd.) and blotted using Vitrobot Mark IV (FEI Co.). Ultra-thin films ($\sim 20 - 200$ nm thick) were formed following removing of excess solution by blotting with filter papers. Specimens were vitrified by rapid plunging into liquid ethane pre-cooled with liquid nitrogen at controlled temperature and relative humidity. The vitrified samples were transferred to a cryo-specimen holder (Gatan model 626; Gatan Inc.) and imaged at −177 °C using a Tecnai G^2 Spirit Twin T-12 TEM (FEI Co.), operated at an acceleration voltage of 120 kV in a

low-dose mode. Images were recorded on a $4K \times 4K$ FEI Eagle CCD camera at defocus values that varied between 2 and $4\,\mu$m.

SAXS Models

SAXS models were calculated using our home-developed data analysis software D+.[17,21] The scattering amplitude, $F^{\text{sol}}_{\text{dimer}}(\vec{q})$, from atomic models of the tubulin dimer subunit in solution were modeled as explained.[2,7,17] The atomic tubulin models were based on PDB ID 5JQG.[22] Missing residues were added according to the published tubulin sequence,[23–25] downloaded from UniProt,[26] and refined using Modeller,[27] as explained.[7] Following refinement, hydrogens were added using MolProbity.[28]

Tubulin assemblies were modeled as fragments of the tubulin single-ring, according to the following symmetry: Chains A and B of PDB 5JQG were translated by vector $\vec{R}_0 = \{x_0, y_0, z_0\} = \{-2.14\,\text{nm}, 8.19\,\text{nm}, 1.73\,\text{nm}\}$ and rotated according to the Tait–Bryan rotation angle convention used in D+,[17,21] where $\{\alpha, \beta, \gamma\} = \{58.16°, -25.15°, -125.65°\}$.

The resulting PDB was placed in a ring symmetry, using the rotation matrix $\mathbf{A}_j\left(0, 0, \gamma_j\right)$,

$$\mathbf{A}_j\left(0,0,\gamma_j\right) = \begin{bmatrix} \cos\left(\gamma_j\right) & -\sin\left(\gamma_j\right) & 0 \\ \sin\left(\gamma_j\right) & \cos\left(\gamma_j\right) & 0 \\ 0 & 0 & 1 \end{bmatrix}$$

which rotates the dimer around the z-axis by γ_j, defined as:

$$\gamma_j = \frac{\pi}{2} - \theta_j$$

for which $\theta_j = {}^{2\pi i}/_{n_{\text{Dimers}}}$, where n_{Dimers} is the number of tubulin dimers in the ring. The

dimer was then translated by:

$$\vec{R}_j = \begin{pmatrix} R_{\text{Ring}} \sin(\theta_j) \\ R_{\text{Ring}} \cos(\theta_j) \\ 0 \end{pmatrix}$$

where R_{Ring} is the ring radius from the origin to the center of mass of the tubulin dimer.

The scattering amplitude of a linear tubulin oligomer, containing n dimers was:

$$F_{\text{n}}(\vec{q}) = \sum_{j=1}^{n} F_{\text{dimer}}^{\text{sol}} \left(\mathbf{A}_j^{-1}\vec{q}\right) \exp\left[i\vec{q} \cdot \vec{R}_j\right]$$

The contribution of the hydration layer of the dimer was taken into account in D+,[17,21] by computing the contribution of voxels around the protein using the following D+ parameters: solvent voxel size of 0.05 nm, solvent probe radius of 0.14 nm, solvation thickness of 0.2 nm, and hydration layer electron density of 364 e/nm^3.[17] The scattering amplitudes of the hydration layer of the ring or its fragments were calculated using the hydrated tubulin dimer subunit and removing the overlapping hydration areas, as described in our earlier publication.[17] To optimize the computation efficiency, all the models were computed after translating their center of mass to the origin.

The radius of ring fragments that best fitted the data was 19.2 nm, whereas the radius of the complete ring that best fitted the data was 18.5 nm.

The modeled scattering intensity of the ring or its linear tubulin oligomer fragments, containing n dimers are:

$$I_{\text{Model,n}}(q) = \left\langle \left| F_{\text{n}}^{\text{hydrated}}(\vec{q}) \right|^2 \right\rangle_{\Omega_q}$$

where, $F_{\text{n}}^{\text{hydrated}}$ is the scattering amplitude of the hydrated F_{n} structure, and $\langle ... \rangle_{\Omega_q}$ represents the orientation averaging of the scattering intensity in reciprocal ($\vec{q}$) space. All the models were then rescaled to an absolute scale, as described.[29]

A Thermodynamic Model of Tubulin Self-Association

To account for the equilibrium between free dimers, curved oligomers, and tubulin single-rings, we have applied a thermodynamic model to determine the mole fractions of the different tubulin assemblies. The derivation of the model was described elsewhere.[7]

The resulting mole fractions, at temperature T and total tubulin concentration $C_{\text{Total}}^{\text{Tubulin}}$, depends on the standard Helmholtz dimer-dimer association free energy per longitudinal contact, $\Delta F_{\text{c}}^{\circ}$, and the standard Helmholtz free energy cost for closing a curved oligomer into a ring, beyond the energy gain from the formation of an additional longitudinal dimer-dimer contract, $\Delta F_{\text{RC}}^{\circ}$.

We have shown[7] that the mole fractions that minimize the total Helmholtz free energy in the grand canonical ensemble are

$$\begin{aligned} X_{\text{n}} &= nX_1^n \exp\left(-\frac{(n-1)\Delta F_{\text{c}}^{\circ}}{k_B T}\right) \\ X_{\text{R}} &= NX_1^N \exp\left(-\frac{\Delta F_{\text{RC}}^{\circ} + N\Delta F_{\text{c}}^{\circ}}{k_B T}\right) \end{aligned} \tag{S1}$$

where k_B is Boltzmann's constant. Solving the conservation of mass,

$$\begin{aligned} &NX_1^N \exp\left(-\frac{\Delta F_{\text{RC}}^{\circ} + N\Delta F_{\text{c}}^{\circ}}{k_B T}\right) \\ &+ \sum_{n=1}^{N} nX_1^n \exp\left(-\frac{(n-1)\Delta F_{\text{c}}^{\circ}}{k_B T}\right) = X_{\text{Total}}^{\text{Tubulin}}, \end{aligned} \tag{S2}$$

for X_1, using the experimental value of $C_{\text{Total}}^{\text{Tubulin}}$ and assuming different values of $\Delta F_{\text{RC}}^{\circ}$ and $\Delta F_{\text{c}}^{\circ}$, result in the entire mole fraction distribution.

The total mole fraction of tubulin dimers in solution was defined as:[30]

$$X_{\text{Total}}^{\prime\text{Tubulin}} = \frac{C_{\text{Total}}^{\text{Tubulin}} N_{\text{AV}}}{M_W^{\text{Tubulin}} \times N_{\text{Total}}}, \tag{S3}$$

where N_{Total} was the total number of molecules in solution ($N_{\text{Total}} = N_{\text{Tubulin}} + N_{\text{Water}} \approx N_{\text{Water}}$), and N_{AV} was the Avogadro number.

To account for stable (kinetically trapped) tubulin rings, that did not disassemble owing to slow kinetics, the total mole fraction of tubulin dimers was defined as:

$$X_{\text{Total}}^{\text{Tubulin}} = \frac{C_{\text{Total}}^{\text{Tubulin}} \left(1 - x_{\text{Stable Rings}}\right) N_{\text{AV}}}{M_W^{\text{Tubulin}} \times N_{\text{Total}}}, \tag{S4}$$

where $x_{\text{Stable Rings}}$ is the mole fraction of stable rings out of the total tubulin concentration:

$$x_{\text{Stable Rings}} = \frac{C_{\text{Stable Rings}}}{C_{\text{Total}}^{\text{Tubulin}}}, \tag{S5}$$

for which $C_{\text{Stable Rings}}$ is the concentration of stable rings in units of mg/mL.

Integrating the Thermodynamic model into the SAXS data analysis

The tubulin oligomers mole fractions in solution were determined by the above thermodynamic model. The expected ensemble scattering intensity was a weighted sum of the oligomers modeled scattering curves, according to the calculated mole fractions:

$$I_{\text{Model}}(q) = C_{\text{Total}}^{\text{Tubulin}} \left(\frac{1}{X_{\text{Total}}^{\prime\text{Tubulin}}} \left(X_{\text{R}} I_{\text{Ring}}(q) + \sum_{n=1}^{\text{N}} X_{\text{n}} I_{\text{Model},n}(q) \right) + x_{\text{Stable Rings}} I_{\text{Stable Ring}}(q) \right). \tag{S6}$$

I_{Ring}, $I_{\text{Stable Ring}}$, and $I_{\text{Model},n}$ were the modeled scattering intensity curves of the closed ring, stable rings, and of a curved oligomer, containing n tubulin dimers, respectively. N is the number of dimers in a full ring. The modeled scattering curves were computed using D+ software (https://scholars.huji.ac.il/uriraviv/book/d-0)[17,21] according to the hydrated atomic model described above, then normalized to absolute units of $^{cm^2}/_{mg}$ (Figure S2).

For each set of $\Delta F_{\text{RC}}^{\circ}$, $\Delta F_{\text{c}}^{\circ}$, and $x_{\text{Stable Rings}}$ values, the total protein concentration, C_{Total}, of the measured sample was fitted by solving Equation S2 for X_1, such that the cost function:

$$\chi^2 = \frac{1}{n_q} \sum_{q=q_{\min}}^{q_{\max}} \left[\frac{I_{\text{Signal}}(q) - \left(I_{\text{Model}}(q) + \text{Const}\right)}{\sigma(q)} \right]^2 \tag{S7}$$

was minimized. n_q is the number of q points within the fitted q-range. Minimizing χ^2 with respect to Const produced the analytic solution, for each experimental scattering curve:

$$\text{Const} = \frac{\sum_{q=q_{\min}}^{q_{\max}} \left[I_{\text{Signal}}(q) - I_{\text{Model}}(q)\right] \sigma(q)^{-2}}{\sum_{q=q_{\min}}^{q_{\max}} \sigma(q)^{-2}}. \tag{S8}$$

$I_{\text{Signal}}(q)$ is the measured scattering intensity, and $\sigma(q)$ is the measured scattering intensity error at a scattering vector whose magnitude is q. $q_{\min}$ and $q_{\max}$ define the q-range used to fit the data, which was set to $0.05 - 3\,\text{nm}^{-1}$, unless otherwise indicated.

Each fit was repeated 10 times, using only one-tenth of the data points, uniformly distributed (within the fitted q-range), and randomly selected. For each fit, the values of χ^2 were computed according to Eqs. S7 and S8 for the entire fitted q-range. A k-means clustering algorithm was then applied on the resulting χ^2 values, to separate the results into two clusters.[12,31] Within the largest cluster, the model with the mean χ^2 value was selected. The errors of the total and the n-th model concentrations were calculated based on the standard deviation around the mean χ^2 value, within the largest cluster.

The global parameters, F°_{RC}, $\Delta F^{\circ}_{\text{c}}$, $C^{\text{Tubulin}}_{\text{Total}}$ and $x_{\text{Stable Rings}}$, were fitted by minimizing χ^2_{Mean} of the concentration series, given by:

$$\chi^2_{\text{Mean}} = \frac{1}{J} \sum_{j}^{J} \chi^2_j \tag{S9}$$

where χ^2_j is the χ^2 value calculated according to Equation S7 for signal j of the concentration series, and J is the number of concentrations measured in the series.

The fitting and clustering algorithms were applied using a Matlab® program, which can be found online (at https://scholars.huji.ac.il/uriraviv/thermodynamic-model-tubulin). .

Kinetic Model of Tubulin Single Ring Assembly/Disassembly

Ring assembly kinetics was assumed to follow a consecutive second-order kinetic steps in the forward direction, and a first-order kinetics in the backward direction. Ring-closing kinetics

was assumed to be first order in both directions.

$$
\begin{gathered}
\mathrm{D_1 + D_1 \underset{k_{-1}}{\overset{k_1}{\rightleftharpoons}} D_2} \\
\mathrm{D_2 + D_1 \underset{k_{-1}}{\overset{k_1}{\rightleftharpoons}} D_3} \\
\vdots \\
\mathrm{D_{12} + D_1 \underset{k_{-1}}{\overset{k_1}{\rightleftharpoons}} D_{13}} \\
\mathrm{D_{13} \underset{k_{-2}}{\overset{k_2}{\rightleftharpoons}} R_{13}}
\end{gathered}
$$

where D_n represents an oligomer containing n tubulin dimers, and R_{13}, corresponds to a tubulin single-ring, containing 13 tubulin dimers. Given k_1 and $\Delta F_\mathrm{c}^\circ$, k_{-1} can be directly calculated from detailed balance:

$$
\begin{gathered}
K_\mathrm{C} = \frac{k_1}{k_{-1}} = \frac{[\mathrm{D}_n]}{[\mathrm{D}_{n-1}]\,[\mathrm{D}_1]} = \\
\frac{N_\mathrm{Total}\frac{X_n}{n}}{N_\mathrm{Total}\frac{X_{n-1}}{n-1}N_\mathrm{Total}\frac{X_1}{1}} = \\
\frac{1}{N_\mathrm{Total}}K_\mathrm{X} = \frac{1}{N_\mathrm{Total}}\exp\left(-\Delta F_\mathrm{c}^\circ\right) \\
\Rightarrow k_{-1} = k_1 \cdot N_\mathrm{Total}\exp\left(\Delta F_\mathrm{c}^\circ\right)
\end{gathered}
\tag{S10}
$$

where K_C and K_X are the equilibrium constants on the concentration and molar fraction scales, respectively. Similarly, k_{-2} can be derived from k_2 and $\Delta F_\mathrm{RC}^\circ$:

$$
\begin{gathered}
\frac{k_2}{k_{-2}} = K_\mathrm{C} = \frac{[\mathrm{R}_{13}]}{[\mathrm{D}_{13}]} = \\
\frac{N_\mathrm{Total}\frac{X_{\mathrm{R}13}}{13}}{N_\mathrm{Total}\frac{X_{13}}{13}} = K_\mathrm{X} = \exp\left(-\Delta F_\mathrm{RC}^\circ\right) \\
\Rightarrow k_{-2} = k_2\exp\left(\Delta F_\mathrm{RC}^\circ\right)
\end{gathered}
\tag{S11}
$$

The series of modeled signals corresponding to the time-resolved experiments, we used the following rate equations:

$$
\begin{gathered}
\frac{d\left[D_n\right]}{dt} = \\
k_1\left[D_1\right]\left(\left[D_{n-1}\right]-\left[D_n\right]\right)+k_{-1}\left(\left[D_{n+1}\right]-\left[D_n\right]\right) \\
\text{for } n \in \{2,...,12\} \\
\frac{d\left[D_{13}\right]}{dt} = \\
k_1\left[D_1\right]\left[D_{12}\right]-k_{-1}\left[D_{13}\right]-k_2\left[D_{13}\right]+k_{-2}\left[R_{13}\right] \\
\frac{d\left[R_{13}\right]}{dt} = k_2\left[D_{13}\right]-k_{-2}\left[R_{13}\right]
\end{gathered} \tag{S12}
$$

The rate equation for $[D_1]$ was calculated from mass conservation:

$$
\frac{d\left[D_1\right]}{dt} = -13\left(\frac{d\left[R_{13}\right]}{dt}\right)-\sum_{n=2}^{13} n\left(\frac{d\left[D_n\right]}{dt}\right) \tag{S13}
$$

This set of ordinary differential equations (ode) was numerically solved to propagate in time the initial conditions, described by the thermodynamic model. The solution was obtained by the Dormand-Prince method (an explicit member of the Runga-Kutta (4,5) family), using the $ode45$ function of Matlab®.

The calculated concentrations were set into Equation S6, following units conversion:

$$
\frac{X_n}{X'^{\text{Tubulin}}_{\text{Total}}} C^{\text{Tubulin}}_{\text{Total}} = nMW_{\text{Tubulin dimer}}\left[D_n\right]_t . \tag{S14}
$$

Kinetic reactions were initiated by a concentration change, a temperature jump, or GTP addition. $\Delta F^\circ_{\text{c}}$ and $\Delta F^\circ_{\text{RC}}$ were fitted to a concentration series of steady-state samples at each condition, and were used to compute the initial state and reverse reactions rate constants, k_{-1} and k_{-2}, according to the thermodynamic model and Equations S10 and S11, respectively.

S2. Cryo-TEM Images

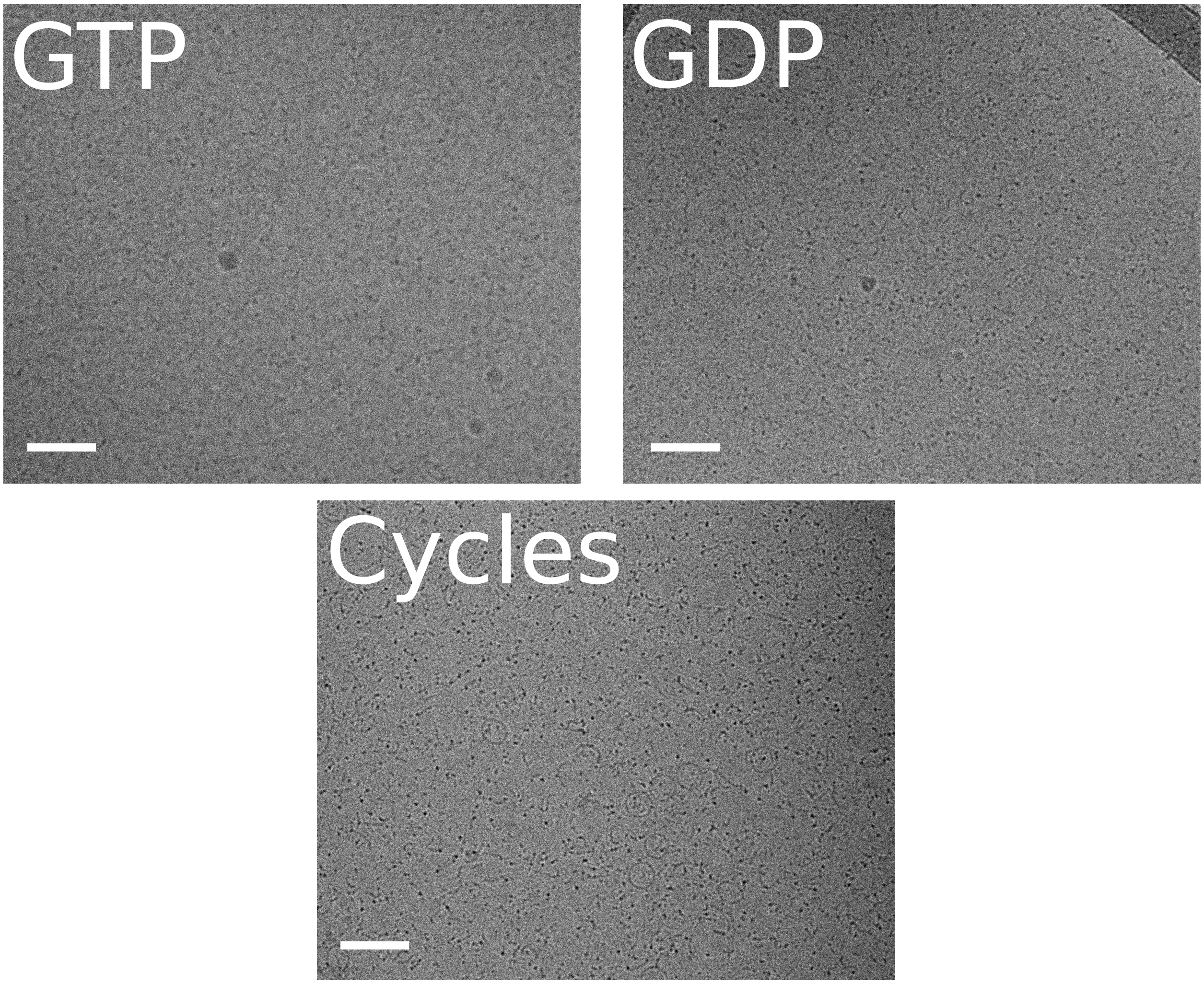

Figure S1: Cryo-TEM images of cold GTP- and GDP-tubulin solutions. GTP and GDP-tubulin were obtained by adding excess GTP and GDP to tubulin solutions, respectively. GDP-tubulin was also obtained after seven heat-cool cycles (see Cryo-TEM). Scale bars equal 100 nm.

S3. SAXS Models of Rings and Ring Fragments

Figure S2 presents the computed solution SAXS curves of ring fragments, containing between one and twelve dimers, as well as tubulin single-rings with two different diameters. Tubulin dimer was modeled according to the atomic model in PDB ID 5JQG, after adding missing residues, as explained.[7]

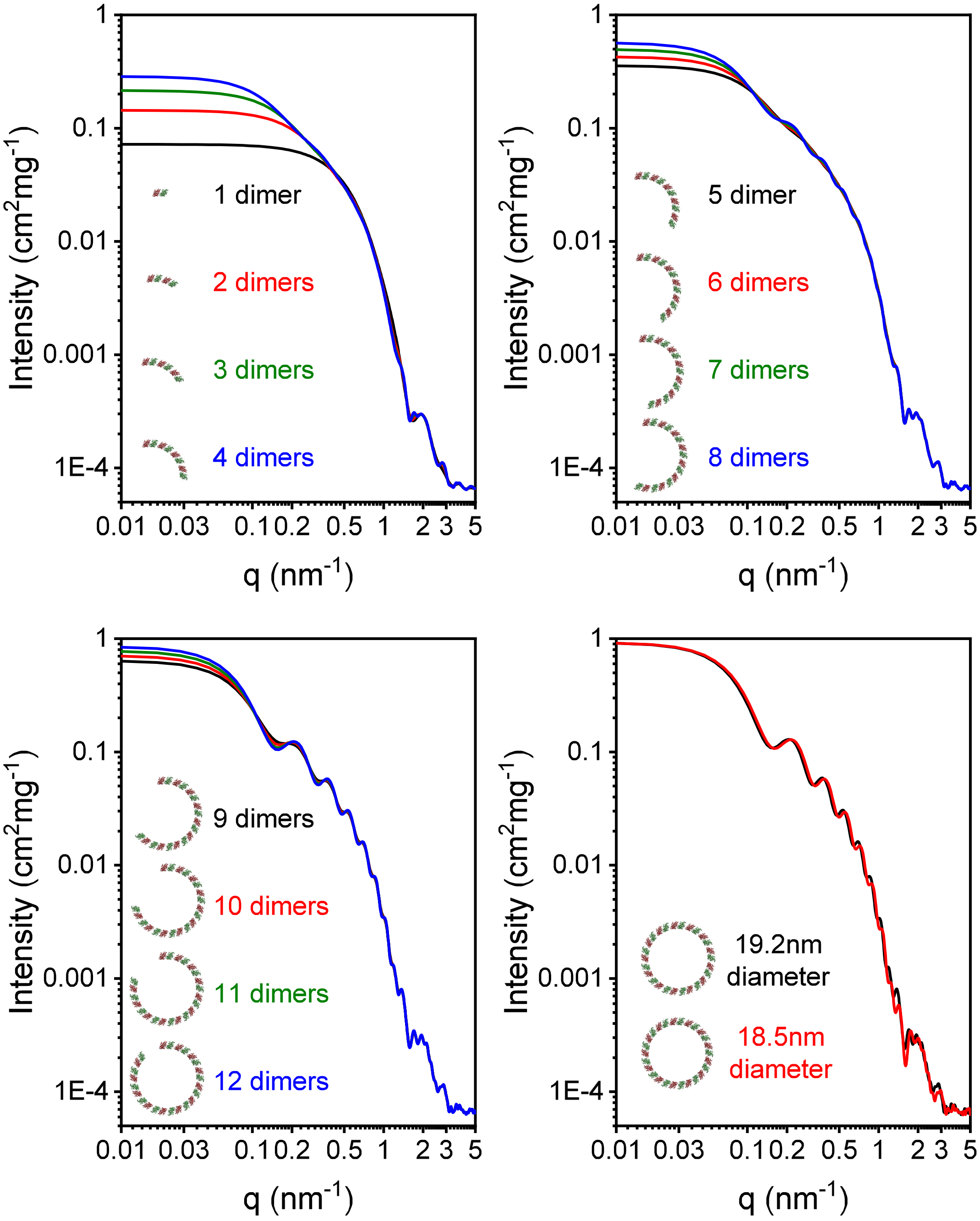

Figure S2: Computed SAXS models of tubulin single-rings and ring fragments used in our data analysis. The atomic tubulin structure, given by PDB ID 5JQG, was docked into the ring or ring fragments assembly symmetry, described in Section SAXS Models. The models were calculated using D+ software.[17,21]

S4. Additional GDP-Tubulin Data

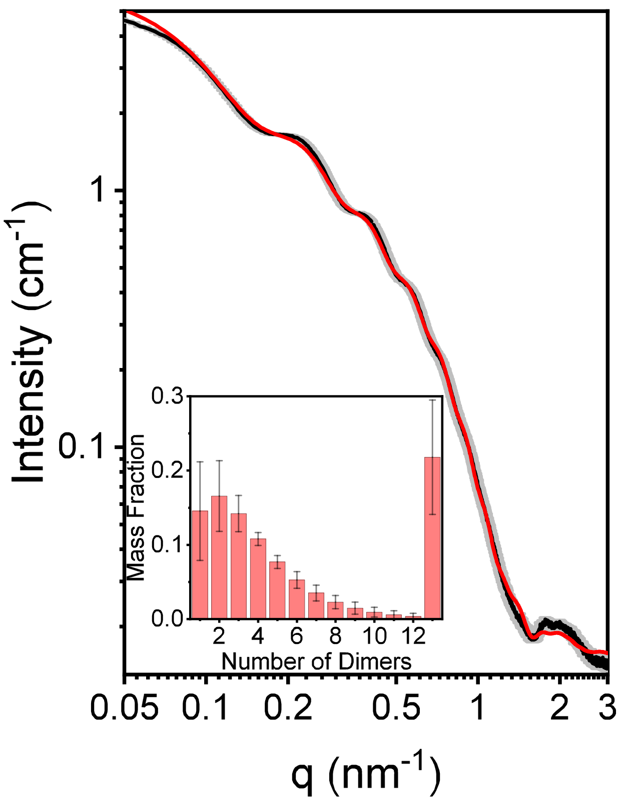

Figure S3: $16 \pm 1\,\mathrm{mg/mL}$ GDP tubulin (obtained by seven heat-cool cycles) were measured by SAXS at 9 °C. The azimuthally integrated background-subtracted scattering data (black curve and gray error bars) were fitted to our thermodynamic model (red curve, computed by Equations S1 and S6). The best-fitted standard Helmholtz free energies were $\Delta F_{\mathrm{c}}^{\circ} = -14.3 \pm 0.5\,\mathrm{k_BT}$ $(-8.0 \pm 0.3\,\mathrm{kcal \cdot mol^{-1}})$ and $\Delta F_{\mathrm{RC}}^{\circ} = 10 \pm 1\,\mathrm{k_BT}$ $(5.6 \pm 0.6\,\mathrm{kcal \cdot mol^{-1}})$, and stable tubulin single-rings comprised 5 % of the total tubulin mass (Equation S4). The inset shows the mass fraction distribution of ring fragments that best-fitted the data as a function of the number of tubulin dimers per oligomer (according to the thermodynamic parameters that best fit the data, Equation S7). Data were measured at the P12 beamline.

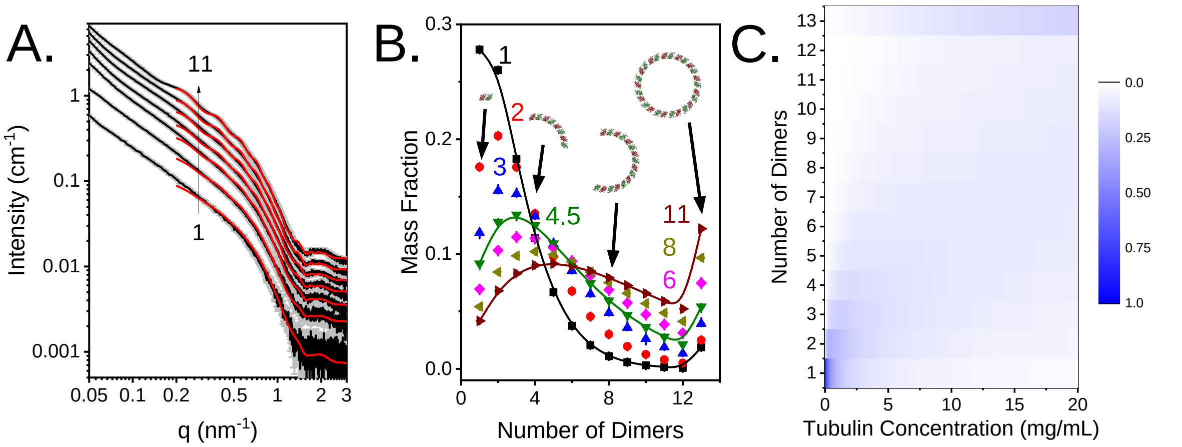

Figure S4: Analysis of a GDP-tubulin concentration series at 36 °C. (A) Tubulin solutions following seven heat-cool cycles, as described in the Sample Preparations Section, and a dilution series, as indicated in the figure, were incubated at 36 °C for 1 h, and measured by SAXS at 36 °C. The azimuthally integrated background-subtracted scattering data (black curves) were fitted to our thermodynamic model (red curves), computed by Equations S1 and S6. The scattering curves of tubulin ring fragments were computed in D+ software,[17,21] by docking the atomic tubulin structure from PDB ID 5JQG into the ring assembly symmetry, as explained in SAXS Models. Figure S2 shows the computed scattering curve of the models used for the analysis. The best-fitted free energy values were $\Delta F^{\circ}_{c} = -16.2 \pm 1\,k_{B}T$ $(-9.9 \pm 0.6\,\mathrm{kcal \cdot mol^{-1}})$ and $\Delta F^{\circ}_{RC} = 16 \pm 1\,k_{B}T$ $(-9.8 \pm 0.6\,\mathrm{kcal \cdot mol^{-1}})$. A short q range $(0.2\,\mathrm{nm^{-1}} < q < 3\,\mathrm{nm^{-1}})$ was used for the curve fitting, due to the severe aggregation, mostly affecting the low q region.[7] Data were measured at the P12 beamline. (B) Mass fraction distribution of the ring fragments that best-fitted the data as a function of the number of tubulin dimers per oligomer (according to the thermodynamic parameters that best fit the data, Equation S7). The numbers indicate the total tubulin concentration in units of mg/mL. (C) Heat map of the tubulin mass fraction plotted in the plane of the number of dimers in assembly versus the total GDP tubulin concentration. The mass fractions were computed according to Equation S1, using the best-fitted thermodynamic parameters.

S5. Steady-State After Ring Disassembly Catastrophe

Following dilution or GTP addition, rapid disassembly and ring catastrophe were observed (Figures 2 and 3). A steady-state was attained after 1 s, and was maintained for at least 57 s following dilution (Figure S5A). After the addition of GTP, steady-state was maintained for at least 12 s (Figure S5B).

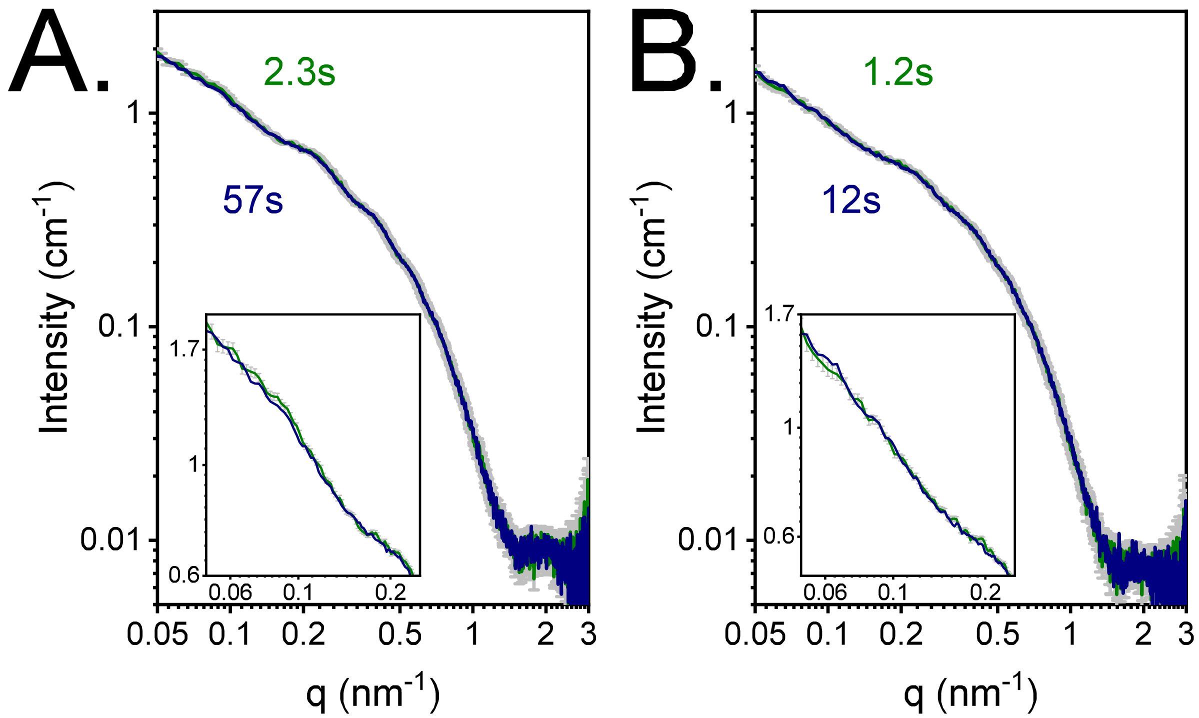

Figure S5: TR-SAXS measurements of GDP-tubulin following rapid dilution or GTP addition at steady-state. GDP tubulin (obtained by seven heat-cool cycles) were mixed in a stopped-flow setup with BRB80, supplemented with either 0.7 ± 0.1 mM GDP (A), or 0.7 ± 0.1 mM GDP and 8 ± 0.5 mM GTP (B), as explained in TR-SAXS Measurement Protocol and Analysis. The similarity between the SAXS curves of the early and late time points in each of the two kinetics suggests that steady-states were rapidly achieved. The time elapsed after the solutions were mixed is indicated in the figures. Experimental error is shown in gray only for the early time points (2.3 s in A and 1.2 s in B). Data were measured at the ID02 beamline.

S6. Tubulin Single Ring Catastrophe Following Dilution

Additional time points from the TR-SAXS data set of Figure 2 and the best-fitted curves, computed according to the kinetic model (Equations S12 and S13).

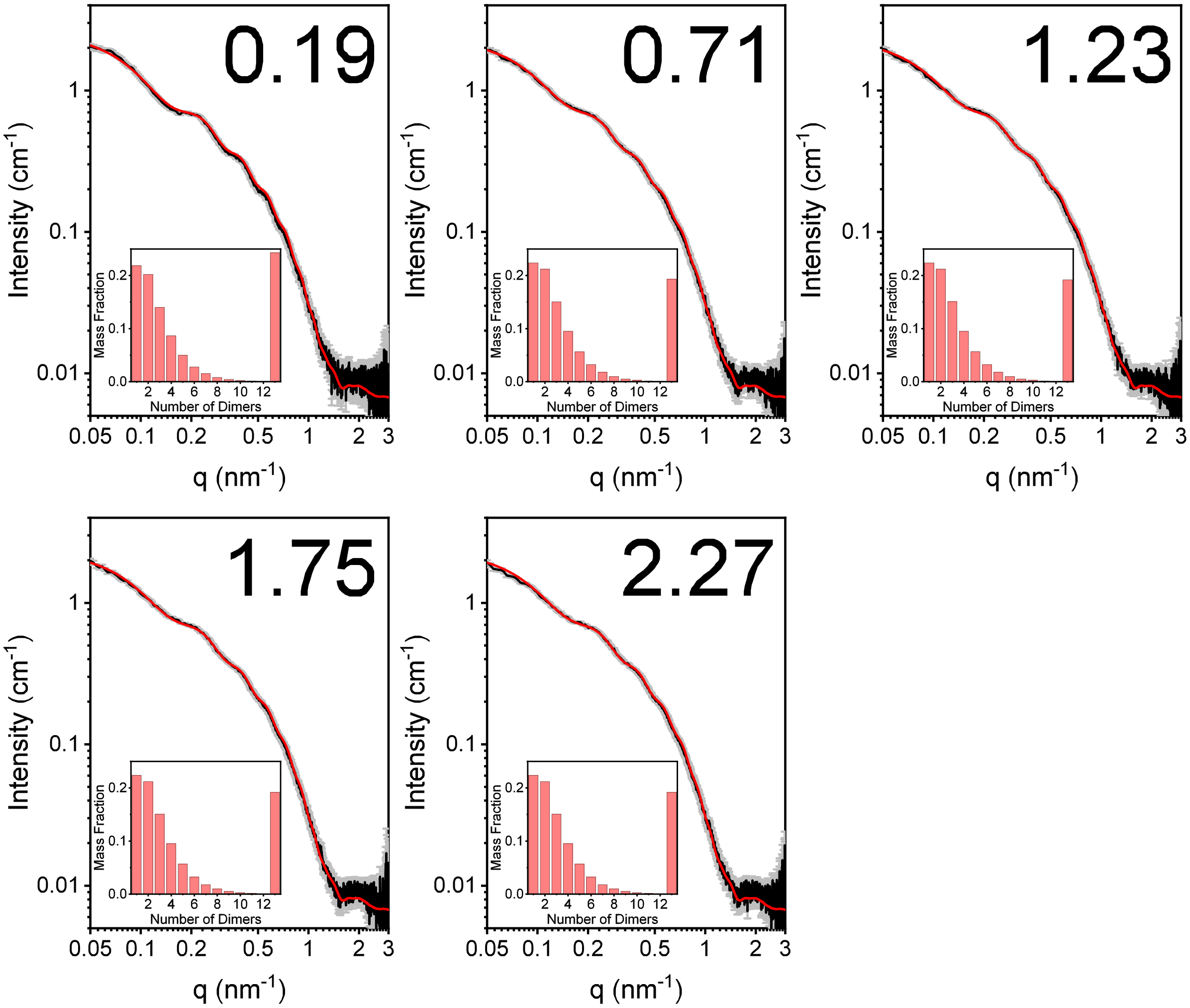

Figure S6: GDP-tubulin single-ring disassembly catastrophe following dilution. GDP tubulin (obtained by seven heat-cool cycles) were mixed in a stopped-flow setup with BRB80, supplemented with $0.7 \pm 0.1\,\mathrm{mM}$ GDP, as explained in TR-SAXS Measurement Protocol and Analysis. TR-SAXS data (black curves, gray error bars) at the indicated time points (in seconds), were fitted (red curves) to our Kinetic Model, as explained in Figure 2. The resulting tubulin mass fractions at each of the tubulin assemblies are shown in Figure 2 and the insets. Data were measured at the ID02 beamline.

S7. Tubulin Single Ring Catastrophe Following GTP Addition

Additional time points from the TR-SAXS data set of Figure 3 and the best-fitted curves, computed according to the kinetic model (Equations S12 and S13).

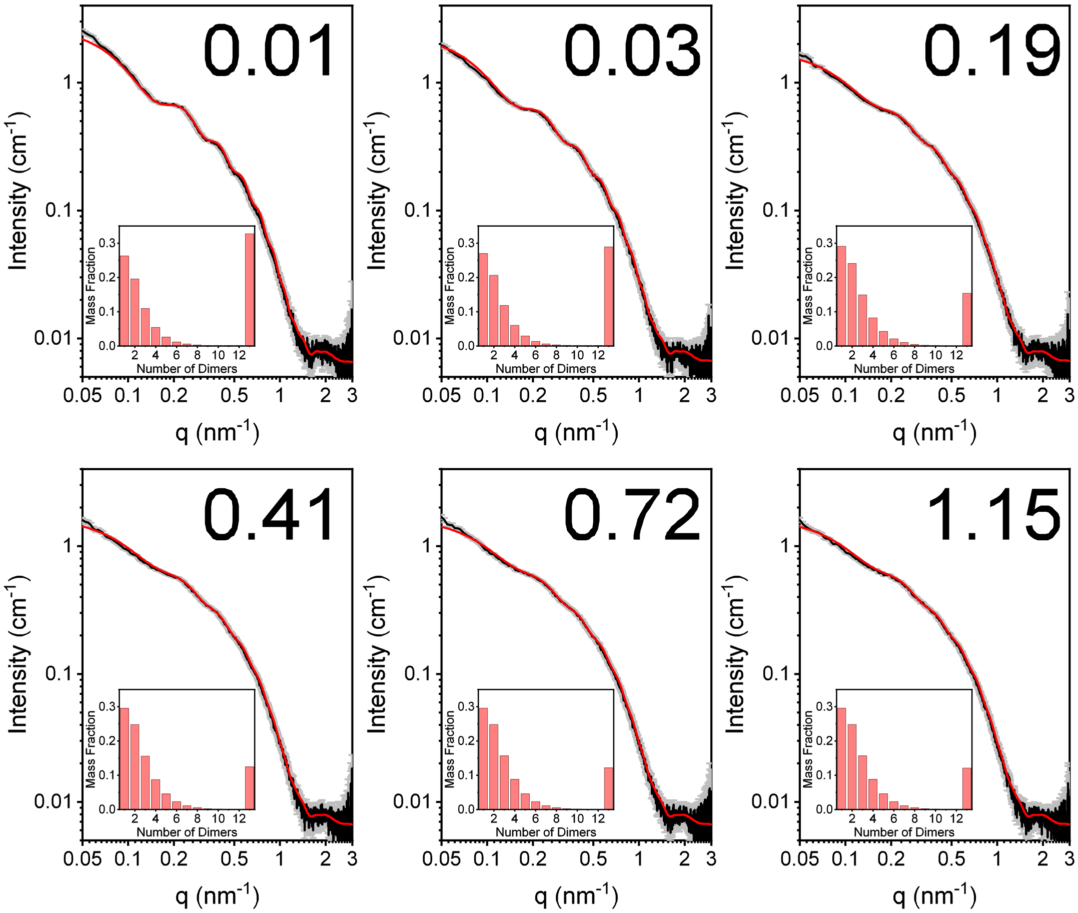

Figure S7: GDP-tubulin single-ring disassembly catastrophe following GTP addition. GDP tubulin (obtained by seven heat-cool cycles) were mixed in a stopped-flow setup with BRB80, supplemented with 0.7 ± 0.1 mM GDP and 8 ± 0.5 mM GTP, as explained in TR-SAXS Measurement Protocol and Analysis. TR-SAXS data (black curves, gray error bars) at the indicated time points (in seconds), were fitted (red curves) to our Kinetic Model, as explained in Figure 3. The resulting tubulin mass fractions at each of the tubulin assemblies are shown in Figure 3 and the insets. Data were measured at the ID02 beamline.

S8. Tubulin Single Ring Disassembly Following a Temperature Increase

Additional time points from the TR-SAXS data set of Figure 4 and the best-fitted curves, computed according to the kinetic model (Equations S12 and S13).

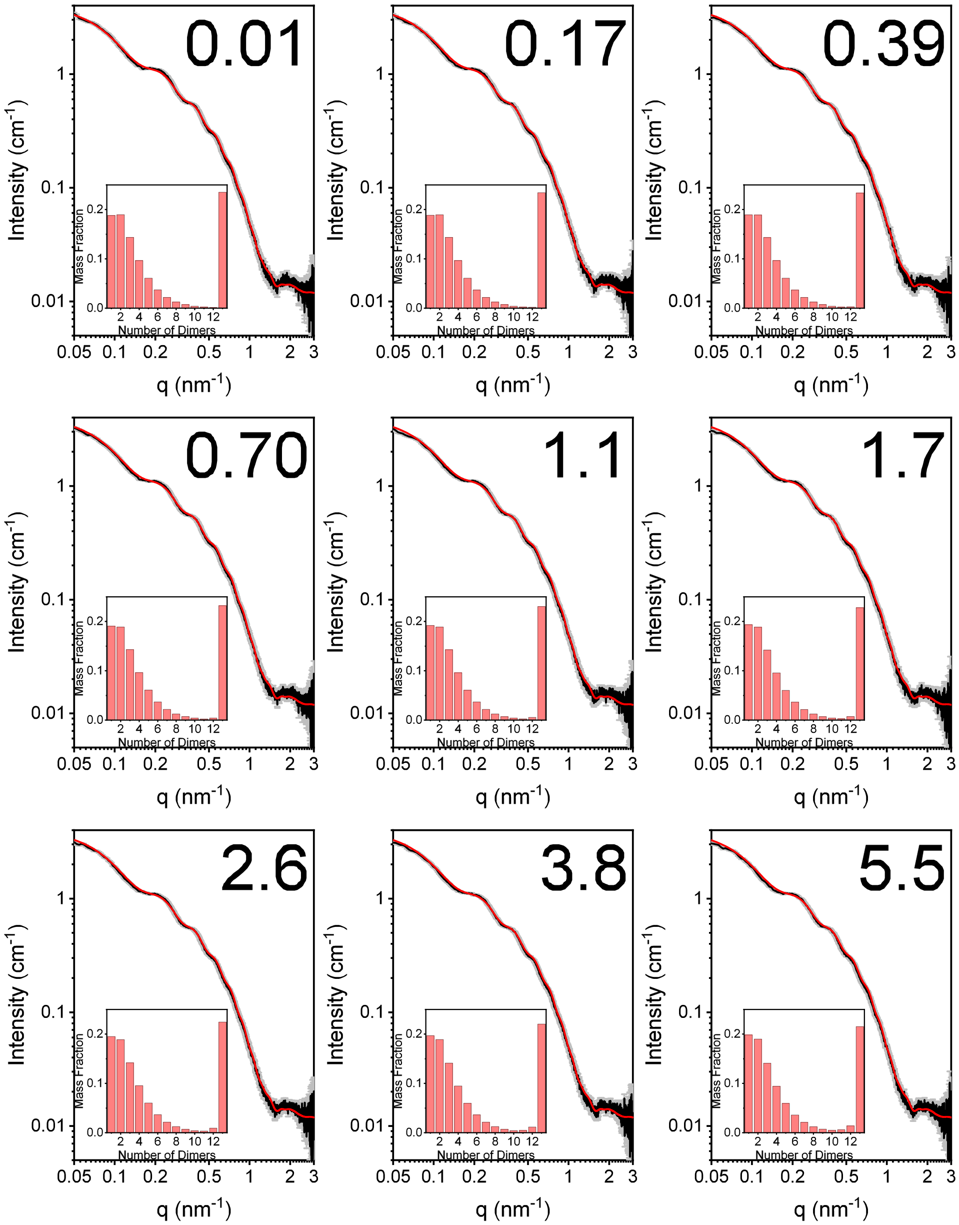

Figure S8: GDP-tubulin single-ring disassembly following a temperature jump. GDP tubulin (obtained by seven heat-cool cycles), kept at 9 °C, were injected by a stopped-flow setup into a quartz capillary, kept at 36 °C, as explained in TR-SAXS Measurement Protocol and Analysis. TR-SAXS data (black curves, gray error bars) at the indicated time points (in seconds), were fitted (red curves) to our Kinetic Model, as explained in Figure 4. The resulting tubulin mass fractions at each of the tubulin assemblies are shown in Figure 4 and the insets. Additional data points are presented in Figure S9. Data were measured at the ID02 beamline.

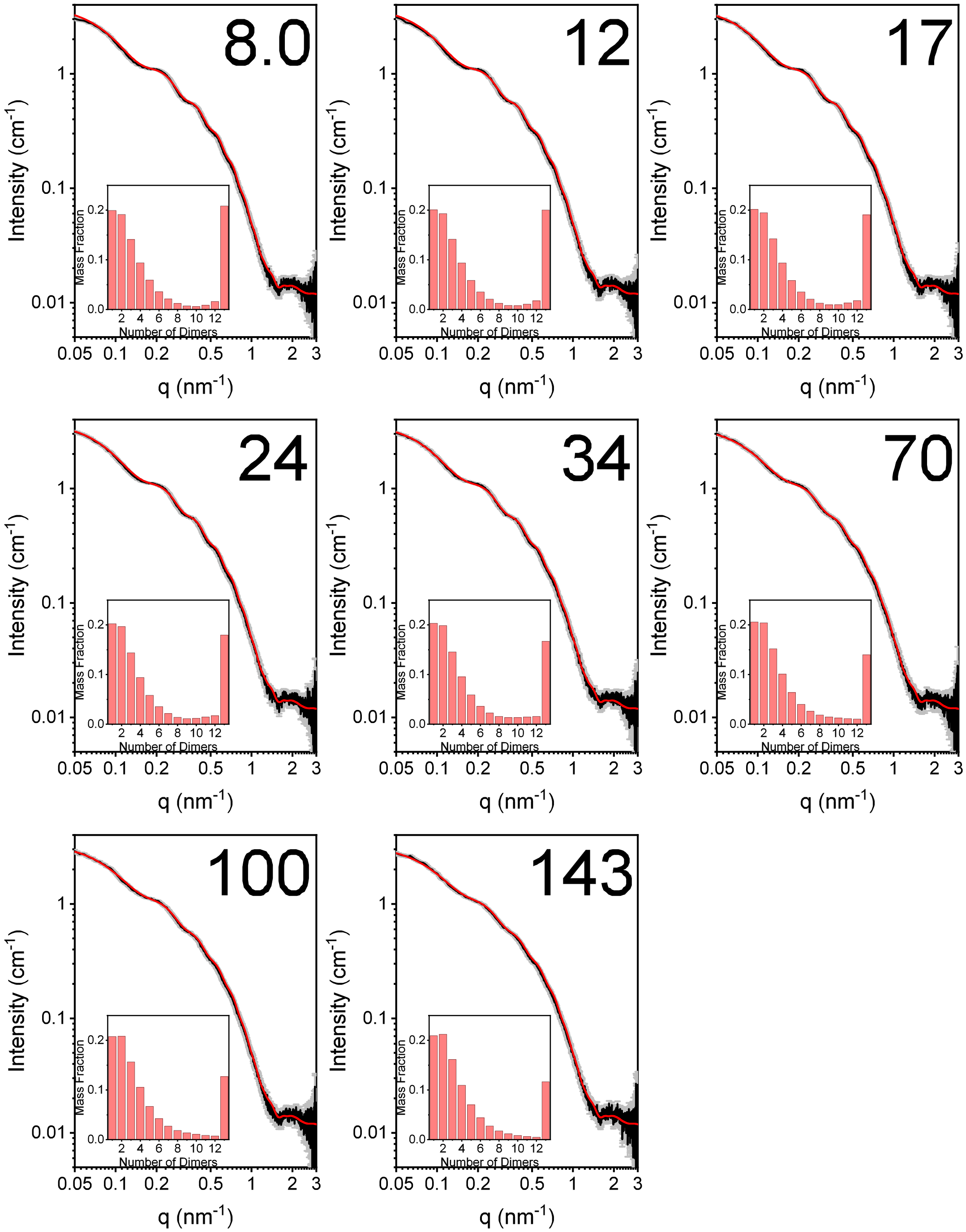

Figure S9: GDP-tubulin single-ring disassembly following a temperature jump; a continuation of Figure S8. The resulting tubulin mass fractions at each of the tubulin assemblies are shown in Figure 4 and in the insets. Data were measured at the ID02 beamline.

References

(1) Castoldi, M.; Popov, A. V. Purification of brain tubulin through two cycles of polymerization-depolymerization in a high-molarity buffer. *Protein Expression Purif.* **2003**, *32*, 83–88.

(2) Shemesh, A.; Ginsburg, A.; Levi-Kalisman, Y.; Ringel, I.; Raviv, U. Structure, assembly, and disassembly of tubulin single rings. *Biochemistry* **2018**, *57*, 6153–6165.

(3) Carlier, M. F.; Pantaloni, D. Kinetic analysis of guanosine 5'-triphosphate hydrolysis associated with tubulin polymerization. *Biochemistry* **1981**, *20*, 1918–1924.

(4) Jacobs, M.; Huitorel, P. Tubulin-associated nucleoside diphosphokinase. *Eur. J. Biochem.* **1979**, *99*, 613–622.

(5) Shemesh Sadeh, A.; Dharan, R.; Ghareeb, H.; Metanis, N.; Ringel, I.; Raviv, U. Effect of tubulin self-association on GTP hydrolysis and nucleotide exchange reactions. *ChemRxiv* **2022**,

(6) Spann, U.; Renner, W.; Mandelkow, E.; Bordas, J.; Mandelkow, E. Tubulin oligomers and microtubule assembly studied by time-resolved X-ray scattering: separation of prenucleation and nucleation events. *Biochemistry* **1987**, *26*, 1123–1132.

(7) Shemesh, A.; Ginsburg, A.; Dharan, R.; Levi-Kalisman, Y.; Ringel, I.; Raviv, U. Structure and energetics of GTP- and GDP-tubulin isodesmic self-association. *ACS Chem. Biol.* **2021**, *16*, 2212–2227, PMID: 34643366.

(8) Blanchet, C. E.; Spilotros, A.; Schwemmer, F.; Graewert, M. A.; Kikhney, A.; Jeffries, C. M.; Franke, D.; Mark, D.; Zengerle, R.; Cipriani, F. et al. Versatile sample environments and automation for biological solution X-ray scattering experiments at the P12 beamline (PETRA III, DESY). *J. Appl. Crystallogr.* **2015**, *48*, 431–443.

(9) Narayanan, T.; Sztucki, M.; Van Vaerenbergh, P.; Léonardon, J.; Gorini, J.; Claustre, L.; Sever, F.; Morse, J.; Boesecke, P. A multipurpose instrument for time-resolved ultra-small-angle and coherent X-ray scattering. *J. Appl. Crystallogr.* **2018**, *51*, 1511–1524.

(10) Round, A.; Felisaz, F.; Fodinger, L.; Gobbo, A.; Huet, J.; Villard, C.; Blanchet, C. E.; Pernot, P.; McSweeney, S.; Roessle, M. et al. BioSAXS sample changer: a robotic sample changer for rapid and reliable high-throughput X-ray solution scattering experiments. *Acta Crystallogr., Sect. D: Biol. Crystallogr.* **2015**, *71*, 67–75.

(11) Kler, S.; Asor, R.; Li, C.; Ginsburg, A.; Harries, D.; Oppenheim, A.; Zlotnick, A.; Raviv, U. RNA encapsidation by SV40-derived nanoparticles follows a rapid two-state mechanism. *J. Am. Chem. Soc.* **2012**, *134*, 8823–8830.

(12) Asor, R.; Schlicksup, C. J.; Zhao, Z.; Zlotnick, A.; Raviv, U. Rapidly forming early intermediate structures dictate the pathway of capsid assembly. *J. Am. Chem. Soc.* **2020**, *142*, 7868–7882.

(13) Asor, R.; Khaykelson, D.; Ben-nun Shaul, O.; Levi-Kalisman, Y.; Oppenheim, A.; Raviv, U. pH stability and disassembly mechanism of wild-type simian virus 40. *Soft Matter* **2020**, *16*, 2803–2814.

(14) Van Vaerenbergh, P.; Léonardon, J.; Sztucki, M.; Boesecke, P.; Gorini, J.; Claustre, L.; Sever, F.; Morse, J.; Narayanan, T. An upgrade beamline for combined wide, small and ultra small-angle x-ray scattering at the ESRF. Proceedings of the 12th International Conference on Synchrotron Radiation Instrumentation (SRI2015). New York, NY, July 6-10, 2015, 2016; p 030034.

(15) Franke, D.; Kikhney, A. G.; Svergun, D. I. Automated acquisition and analysis of small angle X-ray scattering data. *Nucl. Instrum.* **2012**, *689*, 52–59.

(16) Boesecke, P. Reduction of two-dimensional small-and wide-angle X-ray scattering data. *J. Appl. Crystallogr.* **2007**, *40*, s423–s427.

(17) Ginsburg, A.; Ben-Nun, T.; Asor, R.; Shemesh, A.; Fink, L.; Tekoah, R.; Levartovsky, Y.; Khaykelson, D.; Dharan, R.; Fellig, A. et al. *D+*: software for high-resolution hierarchical modeling of solution X-ray scattering from complex structures. *J. Appl. Crystallogr.* **2019**, *52*, 219–242.

(18) Ginsburg, A.; Ben-Nun, T.; Asor, R.; Shemesh, A.; Ringel, I.; Raviv, U. Reciprocal grids: a hierarchical algorithm for computing solution x-ray scattering curves from supramolecular complexes at high resolution. *J. Chem. Inf. Model.* **2016**, *56*, 1518–1527.

(19) Asor, R.; Ben-nun Shaul, O.; Oppenheim, A.; Raviv, U. Crystallization, reentrant melting, and resolubilization of virus nanoparticles. *ACS Nano* **2017**, *11*, 9814–9824.

(20) Orthaber, D.; Bergmann, A.; Glatter, O. SAXS experiments on absolute scale with Kratky systems using water as a secondary standard. *J. Appl. Crystallogr.* **2000**, *33*, 218–225.

(21) Ginsburg, A.; Ben-Nun, T.; Asor, R.; Shemesh, A.; Ringel, I.; Raviv, U. Reciprocal grids: a hierarchical algorithm for computing solution X-ray scattering curves from supramolecular complexes at high resolution. *J. Chem. Inf. Model.* **2016**, *56*, PMID: 27410762.

(22) Yang, J.; Wang, Y.; Wang, T.; Jiang, J.; Botting, C. H.; Liu, H.; Chen, Q.; Yang, J.; Naismith, J. H.; Zhu, X. et al. Pironetin reacts covalently with cysteine-316 of α-tubulin to destabilize microtubule. *Nat. Commun.* **2016**, *7*, 12103.

(23) Wang, H.; Wang, H.; Zhu, Z.; Wu, X.; Yu, M.; Zhao, S.; Yang, S.; Li, K. Full-length cDNA, molecular characterization and physical mapping of five genes from a porcine fetal cDNA library. *Anim. Genet.* **2006**, *37*, 82–84.

(24) Krauhs, E.; Little, M.; Kempf, T.; Hofer-Warbinek, R.; Ade, W.; Ponstingl, H. Complete amino acid sequence of beta-tubulin from porcine brain. *Proc. Natl. Acad. Sci. U.S.A.* **1981**, *78*, 4156–4160.

(25) Linse, K.; Mandelkow, E. M. The GTP-binding peptide of beta-tubulin. Localization by direct photoaffinity labeling and comparison with nucleotide-binding proteins. *J. Biol. Chem.* **1988**, *263*, 15205–15210.

(26) Consortium, U. UniProt: a worldwide hub of protein knowledge. *Nucleic Acids Res.* **2018**, *47*, D506–D515.

(27) Webb, B.; Sali, A. Comparative Protein Structure Modeling Using MODELLER. *Curr. Protoc. Bioinform.* **2016**, *54*, 5.6.1–5.6.37.

(28) Chen, V. B.; Arendall, W. B.; Headd, J. J.; Keedy, D. A.; Immormino, R. M.; Kapral, G. J.; Murray, L. W.; Richardson, J. S.; Richardson, D. C. MolProbity: all-atom structure validation for macromolecular crystallography. *Acta Crystallogr. D: Biol. Crystallogr.* **2010**, *66*, 12–21.

(29) Asor, R.; Selzer, L.; Schlicksup, C. J.; Zhao, Z.; Zlotnick, A.; Raviv, U. Assembly reactions of Hepatitis B capsid protein into capsid nanoparticles follow a narrow path through a complex reaction landscape. *ACS Nano* **2019**, *13*, 7610–7626.

(30) Israelachvili, J. N. In *Intermolecular and surface forces*, 3rd ed.; Israelachvili, J. N., Ed.; Academic Press: San Diego, 2011; pp 503–534.

(31) MacQueen, J. Some methods for classification and analysis of multivariate observations. Proc. Fifth Berkeley Symp. on Math. Statist. and Prob. Berkeley, Calif., 1967; pp 281–297.
