# Supplementary material for: Mechanism of Tubulin Oligomers and Single-Ring Disassembly Catastrophe
Source: J Phys Chem Lett. 2022 Jun 7;13(23):5246–52. doi: 10.1021/acs.jpclett.2c00947 (PMC9208022; doi:10.1021/acs.jpclett.2c00947)
Supplement: Supplementary file 2 — jz2c00947_si_002.pdf [file jz2c00947_si_002.pdf]

jz-2022-00947r.R1

Name: Peer Review Information for "Mechanism of Tubulin Oligomers and Single-Rings Disassembly Catastrophe"

First Round of Reviewer Comments

Reviewer: 1

Comments to the Author

Shemesh et al. have presented outstanding work in assessing the subpopulations of tubulin dimers, tubulin ring fragments, and tubulin rings in cold (depolymerization) conditions using small-angle X-ray scattering (SAXS), building off of earlier modeling by the same group of authors. While this technique would be valuable to the microtubule/cytoskeleton community-at-large, what is really remarkable here is the ability to estimate the Helmholtz free energies from steady-state and TR-SAXS; this should be immediately applicable to scientific communities that look at the evolution of bio or synthetic nanoparticle structures in solution (with a defined end point). While this review recommends publication, there are two questions this reviewer has:

1. In cold conditions, one would expect the number of tubulin dimers in the ring fragments to monotonically decrease (until there are enough dimers to be stabilized within a ring). Why is there an initial increase of dimers in ring fragments at higher tubulin concentration conditions (Fig. 1C)? I'm assuming self-crowding is occurring, but a note about this would be appreciated.

2. Would it be possible to get an estimate for the entropic term?

Reviewer: 2

Comments to the Author

I have read with interest the manuscript from Shemesh et al concerning the mechanisms of disassembly of tubulin oligomers single rings formed by tubulin in the cold.

The manuscript nicely complements the classic SAXS studies of GDP-tubulin polymers by the group of J.M. Andreu J. Mol. Biol. (1994) 238 214- and Biophys J. (1996) 70, 2408- and the thermodynamical ones by Frigon and Timasheff, of course, both the data taken and the computational analysis of the results is largely improved by the method, but still I feel that both the previous studies in the field should have been mentioned and the contradictions between the results obtained and those from the 70´s and 90´s revisited and commented to get a round paper.

The main difference between this manuscript and those of Andreu´s group is the Mg+2 concentration at which the studies are performed, while Andreu´s experiments are done at high Mg+2 concentrations (7 mM) required to form rings and microtubules in absence of PIPES (see also the seminal works of Frigon and Timashef in the 70s) the experiments done by Shemesh et al are done in the presence of los Mg+2 concentrations (1 mM). However, and given the difference of the initial starting points is still impressive the correlation between the temperature induced disassembly kinetics shown in Figures 6 and 7 of Biophys J. (1996) 70, 2408- and the data presented in this manuscript.

In summary I find the manuscript well done, of interest, but in many cases I feel that the seminal works of Frigon and Timasheff in which the thermodynamics of rings formation are described {Frigon, 1975 #46;Frigon, 1975 #47}, and the SAXS characterization of GDP-tubulin aggregates and the kinetics of its disassemble deserve {Diaz, 1994 #405; Díaz, 1996 #481} at least to be mentioned and discussed on the view of these data obtained with new generation synchrotrons and analyzed with computers and not with slide rule or an HP65 :) .

Author's Response to Peer Review Comments:

May 22, 2022

Institute of Chemistry
The Hebrew University of Jerusalem
Jerusalem, Israel 91904
Phone: +972 (2) 658-6030
Email: uri.raviv@mail.huji.ac.il

The Journal of Physical Chemistry Letters

Dear Professor Editor,

Thank you for considering our manuscript entitled "**Mechanism of Tubulin Oligomers and Single-Rings Disassembly Catastrophe**" by Asaf Shemesh, Avi Ginsburg, Raviv Dharan, Yael Levi-Kalisman, Israel Ringel, and Uri Raviv, for publication in *The Journal of Physical Chemistry Letters*.

We thank the two referees for carefully reading our manuscript and for their thoughtful comments. We fully address the comments as explained below.

We are looking forwards to hearing from you.

Best regards,

אורי רביב

Prof. Uri Raviv

Editorial office

1.) TOC Graphic: Provide a TOC image per journal guidelines (2 in x 2 in; on the same page as the abstract) with the heading "TOC Graphic" above the graphic. The graphic should be in the form of a structure, graph, drawing, photograph, or scheme—or a combination. Non-scientific cartoon-like images or caricatures are discouraged. https://pubsapp.acs.org/paragonplus/submission/toc_abstract_graphics_guidelines.pdf

TOC Graphic was added.

2.) References: In both the main file and the supporting information, fix the style of all references to use JPCL formatting (check all references carefully). ***JPC Letters reference formatting requires that journal references should contain: () around numbers, author names, article title (titles entirely in title case or entirely in lower case), abbreviated journal title (italicized), year (bolded), volume (italicized), and pages (first-last). Book references should contain author names, book title (in the same pattern), publisher, city, and year.

References were fixed.

Reviewer 1

1. In cold conditions, one would expect the number of tubulin dimers in the ring fragments to monotonically decrease (until there are enough dimers to be stabilized within a ring). Why is there an initial increase of dimers in ring fragments at higher tubulin concentration conditions (Fig. 1C)? I'm assuming self-crowding is occurring, but a note about this would be appreciated.

Our reply:
In this model, crowding is not playing a role. The difference is owing to the mass fraction scale. At the concentration scale, free dimers are at a higher concentration than tetramers.

In the manuscript, in subsection "Mass fraction Distribution of Tubulin Assemblies", we added the following note:

"At high GDP-tubulin concentrations the mass fraction of dimers in tetramers was higher than the mass fraction of free dimers (Figure 1C). At the concentration scale, however, the concentration of free dimers was higher than the concentration of tetramers, as expected[37]."

2. Would it be possible to get an estimate for the entropic term?

Our reply
We performed the Van 't Hoff analysis and estimated the entropy. In the end of the first paragraph of subsection "Disassembly of GDP-Tubulin Rings Following a Temperature Jump" we added:

"Van 't Hoff analysis, based on the standard self-association free energies at 9 and 36°C (Tables 1 and 2), estimates that upon tubulin self-association the standard entropy increased (by $\approx 12 \pm 10$ cal $\mathrm{K}^{-1}\ mol^{-1}$), suggesting that water molecules were released upon association and increased the entropy of GDP-tubulin oligomerization[49]."

Reviewer 2

The manuscript nicely complements the classic SAXS studies of GDP-tubulin polymers by the group of J.M. Andreu J. Mol. Biol. (1994) 238 214- and Biophys J. (1996) 70, 2408- and the thermodynamical ones by Frigon and Timasheff, of course, both the data taken and the computational analysis of the results is largely improved by the method, but still I feel that both the previous studies in the field should have been mentioned and the contradictions between the results obtained and those from the 70´s and 90´s revisited and commented to get a round paper.

The main difference between this manuscript and those of Andreu´s group is the Mg+2 concentration at which the studies are performed, while Andreu´s experiments are done at high Mg+2 concentrations (7 mM) required to form rings and microtubules in absence of PIPES (see also the seminal works of Frigon and Timashef in the 70s) the experiments done by Shemesh et al are done in the presence of los Mg+2 concentrations (1 mM). However, and given the difference of the initial starting points is still impressive the correlation between the temperature induced disassembly kinetics shown in Figures 6 and 7 of Biophys J. (1996) 70, 2408- and the data presented in this manuscript.

In summary I find the manuscript well done, of interest, but in many cases I feel that the seminal works of Frigon and Timasheff in which the thermodynamics of rings formation are described {Frigon, 1975 #46;Frigon, 1975 #47}, and the SAXS characterization of GDP-tubulin aggregates and the kinetics of its disassemble deserve {Diaz, 1994 #405; Díaz, 1996 #481} at least to be mentioned and discussed on the view of these data obtained with new generation synchrotrons and analyzed with computers and not with slide rule or an HP65 :).

Our reply

The referee is correct. The following penultimate paragraph was added:

"Tubulin double-rings were identified as the depolymerization product of purified MT caused by low temperature[20,21]. The self-association of cold GDP-tubulin into double-rings was examined under excess of 7 mM $MgCl_2$ using velocity sedimentation measurements at increasing tubulin concentration. The observations were described in terms of a thermodynamic model of isodesmic self-association, revealing a longitudinal standard association Helmholtz free energy, ΔF_c°, of ~7.8 kcal/mol[50,51]. TEM and SAXS measurements resolved the structure of the GDP-tubulin double-rings[50,52]. TR-SAXS showed that the double-rings were destabilized within ~ 1 min after the temperature was increased to 37°C or when GTP was added[20,53]."
